# VEGF-C prophylaxis favors lymphatic drainage and modulates neuroinflammation in a stroke model

**DOI:** 10.1084/jem.20221983

**Published:** 2024-03-05

**Authors:** Ligia Simoes Braga Boisserand, Luiz Henrique Geraldo, Jean Bouchart, Marie-Renee El Kamouh, Seyoung Lee, Basavaraju G. Sanganahalli, Myriam Spajer, Shenqi Zhang, Sungwoon Lee, Maxime Parent, Yuechuan Xue, Mario Skarica, Xiangyun Yin, Justine Guegan, Kevin Boyé, Felipe Saceanu Leser, Laurent Jacob, Mathilde Poulet, Mingfeng Li, Xiaodan Liu, Sofia E. Velazquez, Ruchith Singhabahu, Mark E. Robinson, Michael H. Askenase, Artem Osherov, Nenad Sestan, Jiangbing Zhou, Kari Alitalo, Eric Song, Anne Eichmann, Lauren H. Sansing, Helene Benveniste, Fahmeed Hyder, Jean-Leon Thomas

**Affiliations:** 1 Department of Neurology, Yale University School of Medicine, New Haven, CT, USA; 2 Cardiovascular Research Center, Yale University School of Medicine, New Haven, CT, USA; 3 Paris Brain Institute, Université Pierre et Marie Curie Paris 06 UMRS1127, Sorbonne Université, Paris, France; 4 Department of Radiology and Biomedical Imaging, Yale University, New Haven, CT, USA; 5 Department of Biomedical Engineering, Yale University, New Haven, CT, USA; 6 Department of Neurosurgery, Yale University School of Medicine, New Haven, CT, USA; 7 Department of Anesthesiology, Yale School of Medicine, New Haven, CT, USA; 8 Department of Neuroscience, Yale School of Medicine, New Haven, CT, USA; 9 Kavli Institute for Neuroscience, Yale School of Medicine, New Haven, CT, USA; 10 Department of Ophthalmology and Visual Science, Yale University School of Medicine, New Haven, CT, USA; 11 Department of Immunobiology, Yale School of Medicine, New Haven, CT, USA; 12 Paris Cardiovascular Research Center, INSERM U970, Paris, France; 13 Glial Cell Biology Laboratory, Biomedical Sciences Institute, Federal University of Rio de Janeiro, Rio de Janeiro, Brazil; 14 Center of Molecular and Cellular Oncology, Yale Cancer Center, Yale School of Medicine, New Haven, CT, USA; 15 Department of Genetics, Yale School of Medicine, New Haven, CT, USA; 16 Department of Psychiatry, Yale School of Medicine, New Haven, CT, USA; 17 Department of Comparative Medicine, Yale School of Medicine, New Haven, CT, USA; 18 Yale Child Study Center, Yale School of Medicine, New Haven, CT, USA; 19 Program in Cellular Neuroscience, Neurodegeneration and Repair, Yale School of Medicine, New Haven, CT, USA; 20 Faculty of Medicine, Wihuri Research Institute and Translational Cancer Biology Program, University of Helsinki, Helsinki, Finland; 21 Department of Cellular and Molecular Physiology, Yale School of Medicine, New Haven, CT, USA

## Abstract

Meningeal lymphatic vessels (MLVs) promote tissue clearance and immune surveillance in the central nervous system (CNS). Vascular endothelial growth factor-C (VEGF-C) regulates MLV development and maintenance and has therapeutic potential for treating neurological disorders. Herein, we investigated the effects of VEGF-C overexpression on brain fluid drainage and ischemic stroke outcomes in mice. Intracerebrospinal administration of an adeno-associated virus expressing mouse full-length VEGF-C (AAV-mVEGF-C) increased CSF drainage to the deep cervical lymph nodes (dCLNs) by enhancing lymphatic growth and upregulated neuroprotective signaling pathways identified by single nuclei RNA sequencing of brain cells. In a mouse model of ischemic stroke, AAV-mVEGF-C pretreatment reduced stroke injury and ameliorated motor performances in the subacute stage, associated with mitigated microglia-mediated inflammation and increased BDNF signaling in brain cells. Neuroprotective effects of VEGF-C were lost upon cauterization of the dCLN afferent lymphatics and not mimicked by acute post-stroke VEGF-C injection. We conclude that VEGF-C prophylaxis promotes multiple vascular, immune, and neural responses that culminate in a protection against neurological damage in acute ischemic stroke.

## Teaser

Intrathecal VEGF-C pretreatment promotes lymphatic drainage of brain-derived fluids and improves neurological outcomes after ischemic stroke via reduced microglia-mediated neuroinflammation and increased BDNF signaling.

## Graphical Abstract

**Figure:**
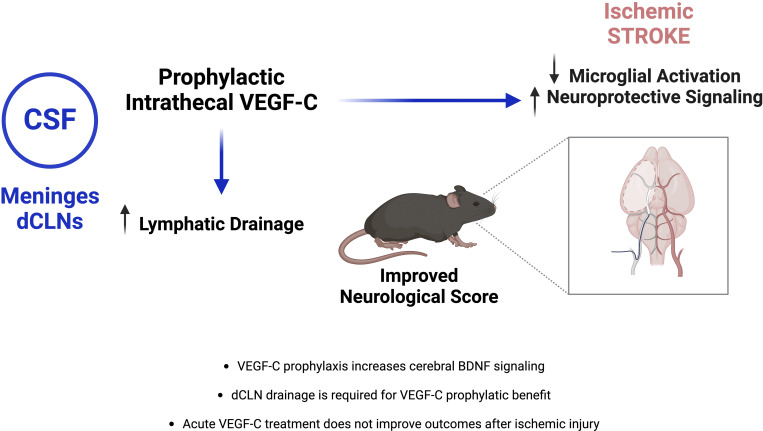

## Introduction

Lymphatic vessels clear metabolic waste, sustain fluid homeostasis, and control the immune surveillance of most body tissues and organs (Aspelund et al., 2016; Oliver et al., 2020). The brain and spinal cord tissues are devoid of lymphatics despite their intense metabolic activity. A large body of research conducted in rodents over the last decade indicates that the clearance of solute waste from central nervous system (CNS) tissues involves cerebrospinal fluid (CSF) flow through the glymphatic system (Bohr et al., 2022), and then through meningeal lymphatic vessels (MLVs) in the dura mater to the CNS-draining CLNs (Proulx, 2021; Rustenhoven and Kipnis, 2022). The regulation of meningeal and pericranial lymphatic drainage is thus of prime importance for the maintenance of brain tissue homeostasis, especially during neuropathological conditions associated with an abnormal accumulation of fluid and waste proteins such as after an acute brain injury.

In mice, CNS-derived fluids can exit the skull and the vertebral column through the cribriform plate and other perineural cranial and vertebral routes and then drain via afferent lymphatics into cervical lymph nodes (CLNs) located in the mandibular, cervical, and lumbosacral regions (Laman and Weller, 2013; Ma et al., 2017a, 2019; Norwood et al., 2019). Recent studies have revealed that MLVs in the dura mater are required for the drainage into CLNs (Antila et al., 2017; Louveau et al., 2018; Ahn et al., 2019; Jacob et al., 2022). The gain or loss of MLVs using a variety of surgical, pharmacological, or gene transfer approaches have been applied to different mouse models of CNS diseases. In acute brain injury, MLVs have been reported to regulate the extent of edema and overall neuronal degeneration in mouse models of brain concussion (Bolte et al., 2020), intracerebral hemorrhage (Tsai et al., 2022), as well as in experimental ischemic injury experiments conducted in zebrafish (Chen et al., 2019) and mice (Yanev et al., 2020). These studies are converging to highlight lymphatics as critical to brain pathology and, therefore, potential therapeutic targets for patients with CNS injuries. However, other reports indicate that inhibition of lymphatics post-ischemic injury may improve the outcomes in murine models of transient middle cerebral artery occlusion (tMCAO) (Esposito et al., 2019; Choi et al., 2023, *Preprint*). Thus, a controversy remains as to how targeting MLVs may benefit patients with ischemic stroke.

Lymphatic development and growth are regulated by vascular endothelial growth factor-C (VEGF-C), which activates the VEGF receptor-3 (VEGFR-3) tyrosine kinase receptor on the surface of lymphatic endothelial cells (LECs) (Lohela et al., 2009). VEGF-C/VEGFR3 signaling controls the growth and plasticity of MLVs, as shown by intrathecal delivery of VEGF-C, either as a recombinant protein or via adeno-associated virus expressing mouse full-length VEGF-C (AAV-mVEGF-C) (Aspelund et al., 2015; Antila et al., 2017; Song et al., 2020; Louveau et al., 2015). Interestingly, VEGF-C is also a proneurogenic factor that activates brain neural stem/progenitor cells (Calvo et al., 2011; Han et al., 2015; Hayakawa et al., 2011; Le Bras et al., 2006). Currently, VEGF-C is the exclusive factor used to stimulate MLVs in preclinical models of brain diseases, but the mechanisms mediating VEGF-C action in the meninges as well as in the brain remain poorly understood.

To gain insight into VEGF-C effects on brain and meningeal cells in mice with ischemic brain injury, we first characterized the consequences of intrathecal delivery of AAV-mVEGF-C in a steady state condition, both on CSF drainage as well as gene expression in the meninges and the brain. VEGF-C overexpression was found to increase CLN drainage in association with MLV remodeling and transcriptomic changes. Single nuclei RNA-sequencing (snRNA-seq) analysis of brain cells revealed activation of a network of VEGF-C upregulated pathways associated with brain-derived neurotrophic factor (BDNF) signaling in subsets of interneurons, astrocytes, and endothelial cells. We next investigated the anatomical and functional outcomes of either VEGF-C prophylaxis prior to acute brain injury or VEGF-C treatment after acute brain injury using the clinically relevant mouse model of focal ischemic stroke induced by tMCAO. We found that VEGF-C prophylaxis, but not post-stroke treatment with VEGF-C-156S, reduced the stroke lesion volume and perilesional inflammation while improving motor behavior. The benefit of VEGF-C prophylaxis was lost upon deep cervical lymph node (dCLN) cauterization, suggesting that it was mainly mediated by VEGF-C/VEGFR-3 signaling in the CSF-draining lymphatic system rather than neural progenitor cells.

Long-term VEGF-C prophylaxis led to a combined increase of CSF lymphatic drainage, reduction of proinflammatory microglial responses, and higher neurotrophic signaling in brain neural cells to counteract the deleterious effects of ischemic injury. In total, the work elucidates the cellular targets of VEGF-C in both control and post-stroke conditions and identifies that long-term elevations in VEGF-C are critical to improve neurological outcomes after stroke.

## Results

### VEGF-C pretreatment promotes CSF drainage to CLNs

**Figure 1.**
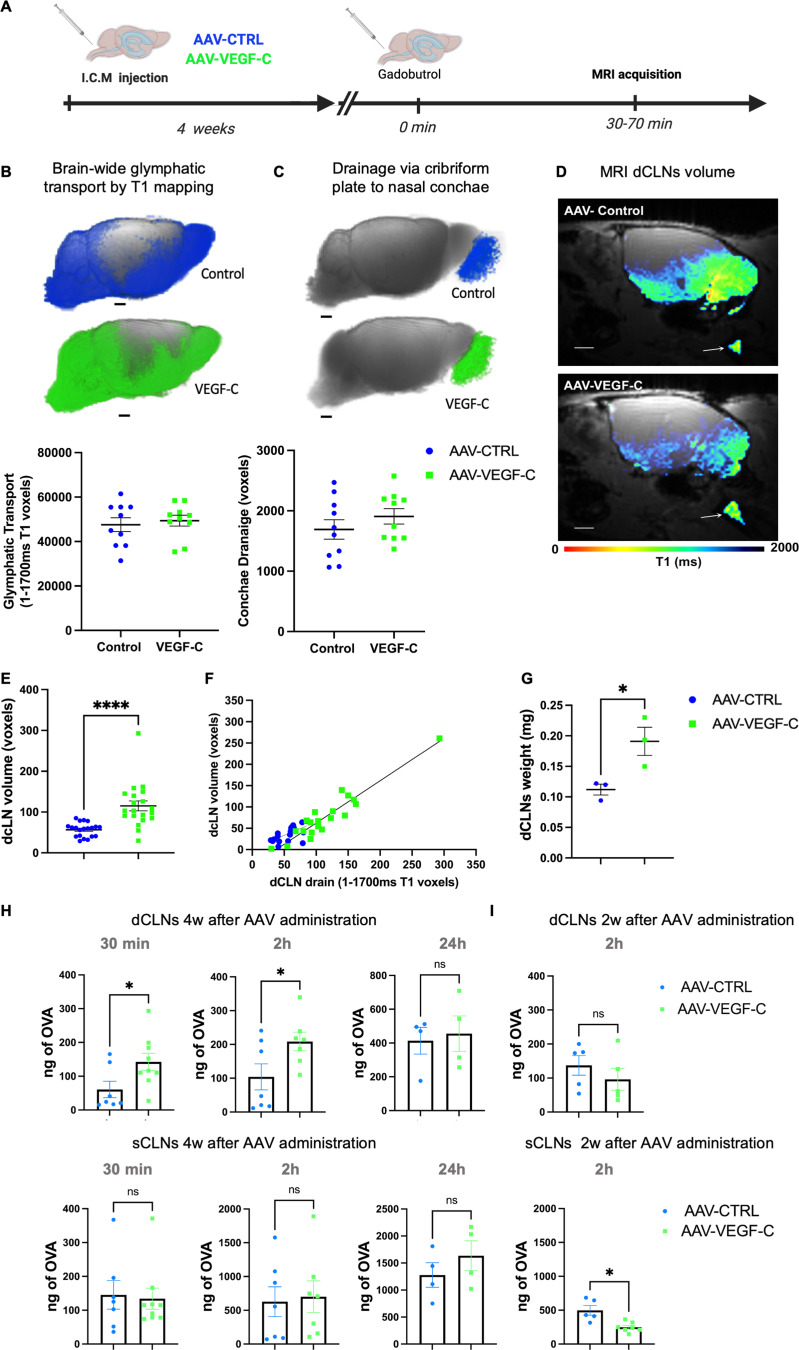
**AAV-mVEGF-C promotes drainage of CSF-injected Gd-DOTA into dCLNs. (A)** Outline of the experimental procedure. 8-wk-old mice were analyzed by DCE-MRI after a prior ICM injection of either AAV-mVEGF-C or AAV-CTRL at 4 wk old (*n* = 10/group). **(B)** T1 mapping (1–1,700 ms range) was performed 1 h after intra-CSF Gd-DOTA administration. Each dot represents one mouse. Note the comparable voxel number between the two groups of mice (P = 0.65, Unpaired *t* test; P = 0.853, Mann–Whitney *U* test). **(C)** T1 mapping of extracranial Gd-DOTA efflux in the cribriform plate–olfactory epithelium area. Each dot represents one mouse (P = 0.30, Unpaired *t* test; P = 0.280, Mann–Whitney *U* test). **(D)** Plane sagittal view of the brain at the level of the dCLNs showing the T1 signal mapped to the node (white arrows) and an enlarged dCLN in the AAV-mVEGF-C mouse compared with CTRL. Note that the nodal volume and the intra-nodal content of Gd-DOTA were both increased in VEGF-C preconditioned mice compared with controls. **(E)** Measurement of dCLN volume through anatomical MRIs (AAV-mVEGF-C: 115.2 ± 12.1 voxels and AAV-CTRL: 56.5 ± 3.7 voxels, *n* = 20 CLNs/group, ****P < 0.0001, Mann–Whitney test). **(F)** Linear regression analysis of data in E (AAV-mVEGF-C: R^2^ = 0.89 P < 0.0001; AAV-control: R^2^ = 0.39 P < 0.004, linear regression model. **(G)** Postmortem analysis of dCLN weight (AAV-mVEGF-C [mg]: 0.19 ± 0.02 and AAV-CTRL [mg]: 0.11 ± 0.01, *P < 0.03, Unpaired *t* test, *n* = 9 dCLNs/group). **(H and I)** Quantification of ICM-injected fluorescent OVA-A^647^ in dCLNs and sCLNs at indicated timepoints 4 wk (H) and 2 wk (I) after AAV administration (*n* = 4–9/group, *P < 0.05, Mann–Whitney test). Data are represented as mean ± SEM. Scale bar: 1.5 mm (B–D). w, week.

To assess the consequences of intrathecal AAV-mVEGF-C delivery on the glymphatic-lymphatic system in vivo, we conducted quantitative magnetic resonance imaging (MRI). Mice received an intra-cisterna magna (ICM) injection of AAV-mVEGF-C or AAV encoding soluble mVEGFR3_4–7_-Ig (VEGFR-3 ectodomains that do not bind VEGF-C) (AAV-CTRL) (Fig. 1 A). 4 wk after treatment, we performed an MRI using T1 mapping 1 h after ICM administration of gadoteric acid (Gd-DOTA, molecular weight: 558 Da) (Koundal et al., 2019; Xue et al., 2020). All MRI studies were carried out on a 9.4T MRI instrument on ketamine/xylazine anesthetized mice.

Analysis of T1 mapping data showed that brain-wide glymphatic transport of Gd-DOTA was unaffected by VEGF-C treatment compared with controls (Fig. 1 B, P = 0.65). In the same mice, we also evaluated drainage of Gd-DOTA via cranial outflow pathways. Quantification of Gd-DOTA efflux to the nasal cavity via the cribriform plate did not reveal any differences in efflux between control and VEGF-C treated mice (P = 0.30) (Fig. 1 C).

Next, we evaluated the drainage of Gd-DOTA to the dCLNs using the quantitative T1 map analysis of Gd-DOTA uptake (Fig. 1 D). Anatomical MRI showed that there was no difference between left and right dCLN volume in control mice (left 0.35 ± 0.09 mm^3^ versus right 0.32 ± 0.11 mm^3^, P = 0.434). Most notably, the dCLN volume was significantly increased in VEGF-C–pretreated mice when compared with controls (dCLN volume AAV-CTRL: 0.34 ± 0.10 mm^3^ versus dCLN volume AAV-mVEGF-C: 0.69 ± 0.32 mm^3^, ****P < 0.0001). Furthermore, we found a statistically significant increase in dCLN drainage in VEGF-C–pretreated mice compared with controls (Fig. 1 E). A linear regression analysis showed that dCLN volume was directly related to Gd-DOTA uptake into the dCLNs and enhanced by VEGF-C (Fig. 1 F). Finally, postmortem analysis further validated that the VEGF-C pretreatment increased the weight of dCLNs (Fig. 1 G).

Next, we examined the histology of CLNs after ICM injection of a fluorescent tracer (OVA-A^647^). Mice were treated for 4 wk with AAV-CTRL and AAV-mVEGF-C, and then OVA-A^647^ content was measured at 30 min, 2, or 24 h after OVA-A^647^ injection. At 30 min and 2 h after tracer injection, OVA-A^647^ content in dCLNs of VEGF-C–treated mice was increased compared with controls; this effect was no longer observed at 24 h (Fig. 1 H). The superficial nodes (sCLNs) showed much higher OVA-A^647^ content compared with dCLNs but no significant increase in tracer drainage after VEGF-C treatment (Fig. 1 H). We detected no change in dCLN drainage after 2 wk of VEGF-C treatment, indicating that >2 wk of VEGF-C action is required to improve dCLN drainage (Fig. 1 I). Altogether, the MRI and histological data provide converging evidence that VEGF-C increases CSF outflow into the dCLNs over time.

### VEGF-C promotes growth and transcriptomic changes in CSF-draining lymphatics

**Figure 2.**
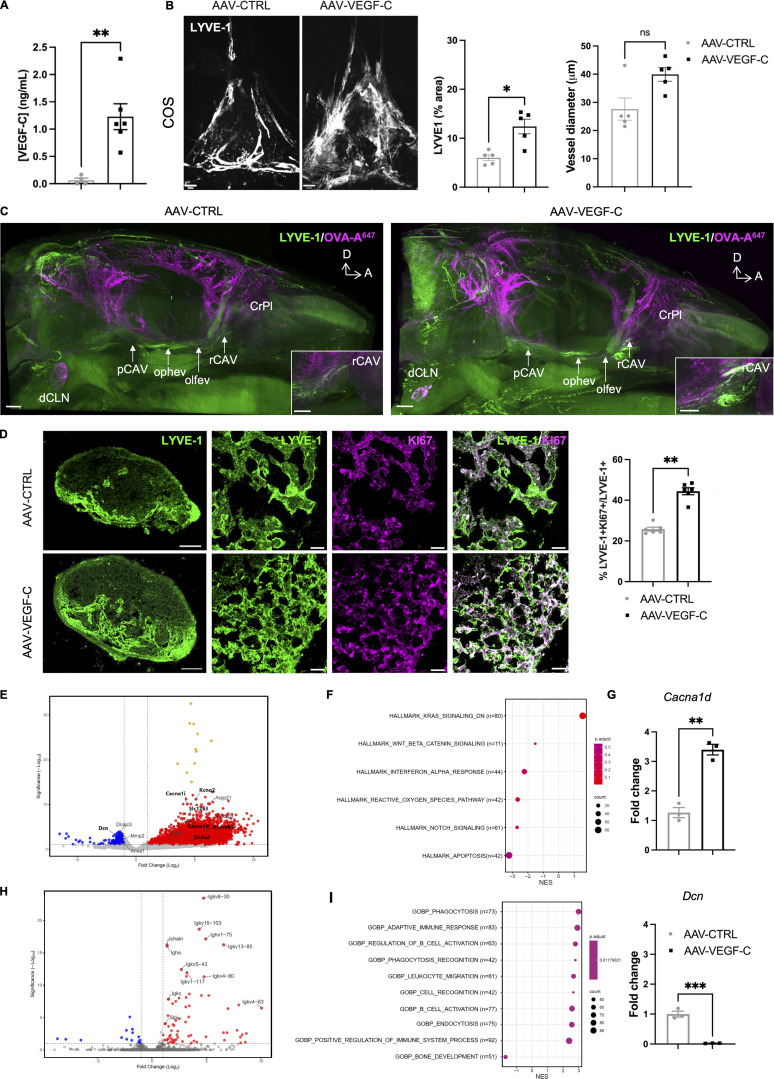
**VEGF-C effects in CSF-draining lymphatics. (A)** VEGF-C protein expression measured by ELISA of the CSF (*n* = 4–6 mice/group, **P < 0.01, Mann–Whitney test). **(B and C)** MLV immunolabeling. **(B)** Confocal imaging of MLVs labeled with the indicated antibody in the confluence of sinuses (COS) and quantification of MLV surface and diameter (*n* = 5 mice/group, *P < 0.05, Mann–Whitney test). **(C)** LSFM imaging of mice treated as indicated for 4 wk, injected with OVA-A^647^ 30 min before sacrifice, then stained with anti-LYVE-1 antibody (*n* = 3 mice/group). Posterior cavernous sinus (pCAV), ophthalmic emissary vein (ophev), olfactory emissary vein (olfev), rostral cavernous sinus (rCAV), and cribriform plate (CrPl). Insets show higher magnifications of OVA-A^647^ uptake into the rCAV (white). **(D)** dCLN immunolabeling of LYVE-1^+^ and KI67^+^ cells in mice treated for 4 wk with either AAV-mVEGF-C or -CTRL. Quantification of KI67^+^LYVE-1^+^/LYVE-1^+^ pixel area. Scale bar: 100 μm (left panel) and 20 μm (right panels). **(E–G)** Bulk RNA-seq analysis of FACS-sorted dural LECs. Volcano plot of DEGs between CTRL and VEGF-C–treated mice in mRNA extracted from dural LECs. Downregulated genes (blue) and upregulated genes (red). **(F)** HALLMARK GSEA dot-plot depicting the most upregulated and downregulated pathways in the AAV-mVEGF-C group compared to control. N = number of DEGs/pathway. NES, normalized enrichment score. **(G)** qPCR analysis of dura mater mRNAs. Expression of indicated genes in the AAV-mVEGF-C group compared with controls (*n* = 3 mice/group, **P < 0.01, ***P < 0.005 Mann–Whitney test). **(H)** Volcano plot of DEGs between CTRL- and VEGF-C–treated mice in mRNA from FACS-isolated dural CD45^+^ leukocytes. Downregulated genes (blue) and upregulated genes (red). **(I)** GSEA dot plot based on the GO biological process (GOBP) illustrating the most upregulated and downregulated pathways in the AAV-mVEGF-C group compared with control. Scale bar: 600 μm (A); 1,000 μm (C); 100 μm (D, left panel); 20 μm (D, right panels).

**Figure S1.**
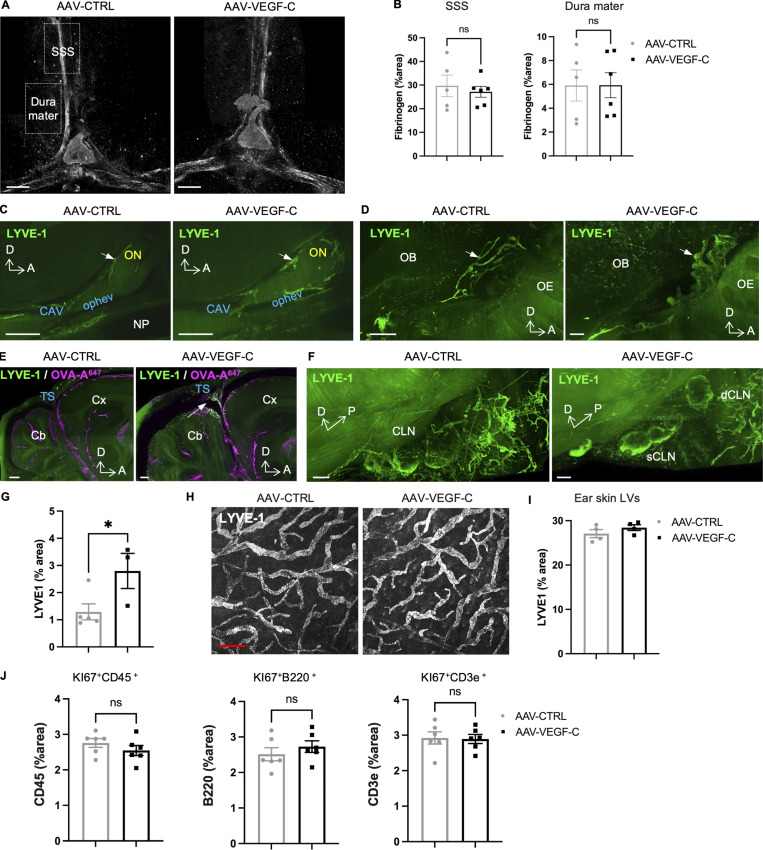
**Effects of VEGF-C prophylaxis on the dural vasculature and CLN. (A and B)** Confocal imaging (A and B) quantification of fibrinogen labeling in the dura mater superior sagittal sinus (SSS) and in the neighboring dura mater (boxed areas in A) (*n* = 5–6 mice/group). **(C–F)** LSFM imaging of LYVE-1^+^ cells in the meninges (C–E) and CLN (F). LYVE-1^+^ MLV located along the olfactory nerve (ON, white arrow) and the cavernous sinus at the level of the ophthalmic emissary vein (ophev) (C) and close to the cribriform plate (white arrow) (D). OB: olfactory bulb; OE: olfactory epithelium. Note the expansion of MLV coverage upon VEGF-C prophylaxis. **(E)** Pial and brain perivascular OVA-A647^+^/LYVE-1^+^ phagocytic cells (white arrow) were observed in the cerebellar region of VEGF-C–treated mice 30 min after ICM injection of OVA-A647. **(F)** LYVE1 expression in sCLNs and dCLNs (see also Fig. 1 D). **(G)** Quantification of confocal images of LYVE-1^+^ lymphatics in the lower region of the olfactory mucosa. *P < 0.05. **(H and I)** Representative images (H) and quantification (I) of LYVE-1^+^ immunostaining in the ear skin (*n* = 4 mice/group). **(J)** Quantification of KI67^+^ immune cells among leukocytes (CD45^+^), B lymphocytes (B220^+^), and T lymphocytes (CD3e^+^) in the dCLNs (*n* = 6 mice/group). Scale bar: 600 μm (A); 1,000 μm (C–F); 50 μm (H).

VEGF-C levels in the CSF increased after 4 wk of AAV-mVEGF-C delivery, as measured by ELISA (Fig. 2 A). VEGF-C induced an increase in the area covered by lymphatic vessels expressing lymphatic vessel endothelial hyaluronan receptor 1 (LYVE-1) in the dorsal dura when compared with controls, without significantly increasing LYVE-1^+^ lymphatic vessel diameter (Fig. 2 B). In agreement with the previous observation that AAV-mVEGF-C does not affect blood vessels (Antila et al., 2017), we found no leakage of fibrinogen from dura blood vessels in AAV-mVEGF-C–treated mice (Fig. S1, A and B). Light sheet fluorescence microscopy (LSFM) imaging of iDISCO^+^-cleared whole heads revealed that VEGF-C induced enlargement of dural LYVE-1^+^ lymphatics at the anterior skull base, around the cavernous sinus, and of extracranial lymphatics interconnected with the cavernous sinus (Fig. 2 C, Fig. S1, C–F; and Videos 1 and 2). An increased number of phagocytic LYVE-1^+^ pial and perivascular cells were also observed in VEGF-C–treated mice (Fig. S1, E). Quantification of confocal images showed that VEGF-C increased the growth of olfactory mucosa lymphatics (Fig. S1 G), but not of lymphatics in the ear skin (Fig. S1, H and I). In the dCLN, VEGF-C increased LEC proliferation measured by anti-Ki67 labeling on cryosections (Fig. 2 D) but did not affect cell numbers of CD45^+^, CD3a^+^, and B220^+^ leukocytes (Fig. S1 J). Therefore, intrathecal VEGF-C expands lymphatics in all compartments of the CSF drainage pathway, that is, in the dura mater, the extracranial tissues, and the CLNs, but not the ear skin.

**Video 1.** **Lateral LSFM views of the head of an adult mouse injected into the cisterna magna with AAV-CTRL at 1 mo prior to euthanasia.** LYVE-1 staining (green) labels lymphatic vessels and perivascular macrophages; OVA-A^647^ tracer (magenta) has been phagocyted by macrophages in the brain meninges and, outside of the skull, in the nasal cavity and the LNs. Note MLVs along the caudal part of the cavernous sinus. The frame rate is 50 frames/s.

**Video 2.** **Lateral LSFM views of the head of an adult mouse injected into the cisterna magna with AAV-VEGF-C at 1 mo prior to euthanasia.** LYVE-1 staining (green) labels lymphatic vessels and perivascular macrophages; OVA-A^647^ tracer (magenta) has been phagocyted by macrophages in the brain meninges and, outside of the skull, in the nasal cavity and the LNs. Note expanded MLVs along the rostral part of the cavernous sinus.The frame rate is 50 frames/s.

**Figure S2.**
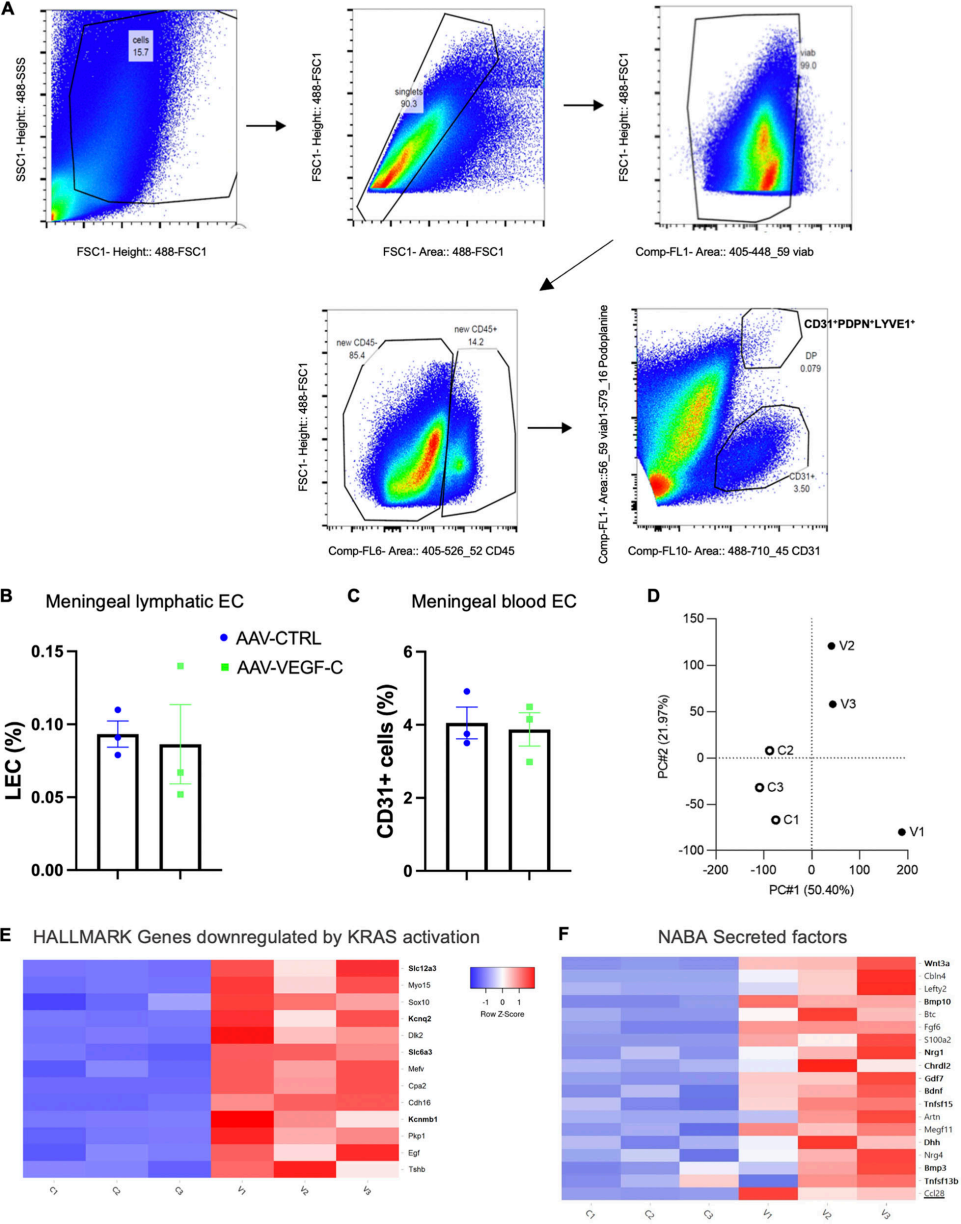
**Isolation and bulk RNA-seq analysis of dural LECs and immune cells. (A)** Flow cytometry gating strategy for isolating LYVE-1^+^/PDPN^+^/CD31^+^/CD45^−^ LECs from the dorsal dura mater of AAV-CTRL– and AAV-VEGF-C–treated mice (*n* = 30 mice/group; two groups; three independent experiments). **(B and C)** Percentage of dural LECs (B) and blood ECs (C) in AAV-VEGF-C and -CTRL mice. **(D)** Principal component analysis segregation of LEC transcripts from AAV-CTRL and -VEGF-C mice. **(E)** HALLMARK gene set of the uppermost upregulated signaling pathway by VEGF-C. **(F)** NABA set of the 18 most expressed genes in the secreted factor signaling pathway, the uppermost upregulated up by VEGF-C. Note expression of osteogenic/osteoclastic factors and chemokine CCL28 (underlined).

To gain molecular insight into the response of dural lymphatics to VEGF-C, we isolated LYVE-1^+^/Podoplanin^+^(PDLN^+^)/CD31^+^/CD45^−^ LECs from the dorsal dura mater of AAV-CTRL– and AAV-mVEGF-C–treated mice (Fig. S2 A). We obtained similar numbers of LECs and CD31^+^ LYVE-1-negative blood endothelial cells from both groups (Fig. S2, B and C). Bulk RNA-seq analysis of dural LECs followed by principal component analysis showed a clear segregation of LEC transcripts from CTRL- and VEGF-C–treated mice (Fig. S2 D). The volcano plot of differentially expressed genes (DEGs) between CTRL- and VEGF-C–treated mice showed that the top 10 genes upregulated by VEGF-C belonged to the signaling pathway downregulated by KRAS (KRAS-DN), the most highly upregulated HALLMARK pathway (*n* > 80 genes, Fig. 2 E and Fig. S2 E). KRAS-DN included genes encoding the *Kcnq2* potassium voltage-gated channel (logFC [fold change] = 5.10, false discovery rate [FDR] = 6.98 × 10^−12^), the *Cacna1* calcium voltage-gated channel gene (logFC = 4.37, FDR = 8.83 × 10^−1^), the *Slc12a3* and *Slc6a3* sodium-chloride cotransporters (logFC = 4.76, FDR = 4.89 × 10^−3^; logFC = 4.55, FDR = 1.73 × 10^−8^), as well as the potassium calcium-activated channel *Kcnmb1* gene (logFC = 5.91, FDR = 7.06 × 10^−5^). Signaling pathways highly (*n* > 40 genes) downregulated by VEGF-C in dural LECs included interferon α (IFN-α) response and apoptosis (Fig. 2 F). Quantitative PCR (qPCR) analysis of dura mater from CTRL- and VEGF-C–treated mice confirmed transcriptomic data, specifically that VEGF-C significantly increased *Cacna1d* expression and decreased *Decorin* expression (Fig. 2 G). In the NASA GeneLab RNA-seq consensus pipeline, the top upregulated pathway encoded secreted factors activated by VEGF-C (*n* = 77 genes, P = 0.0026), including many upregulated osteogenic-osteoclastic factors, and CCL28 (logFC = 4.06, FDR = 9.82 × 10^−5^), a chemokine involved in mucosal immunity (Hieshima et al., 2003) (Fig. S2 G).

Complementary RNA-seq analyses were carried out on dural immune cells (Fig. S2 A). VEGF-C was found to increase expression of genes associated with phagocytic activity and activation of adaptive B cell responses (Fig. 2 H), including the upregulation of numerous immunoglobulin (IgG and IgA) genes (Fig. 2 I).

### VEGF-C induces a neuroprotective transcriptomic signature in brain tissues

**Figure S3.**
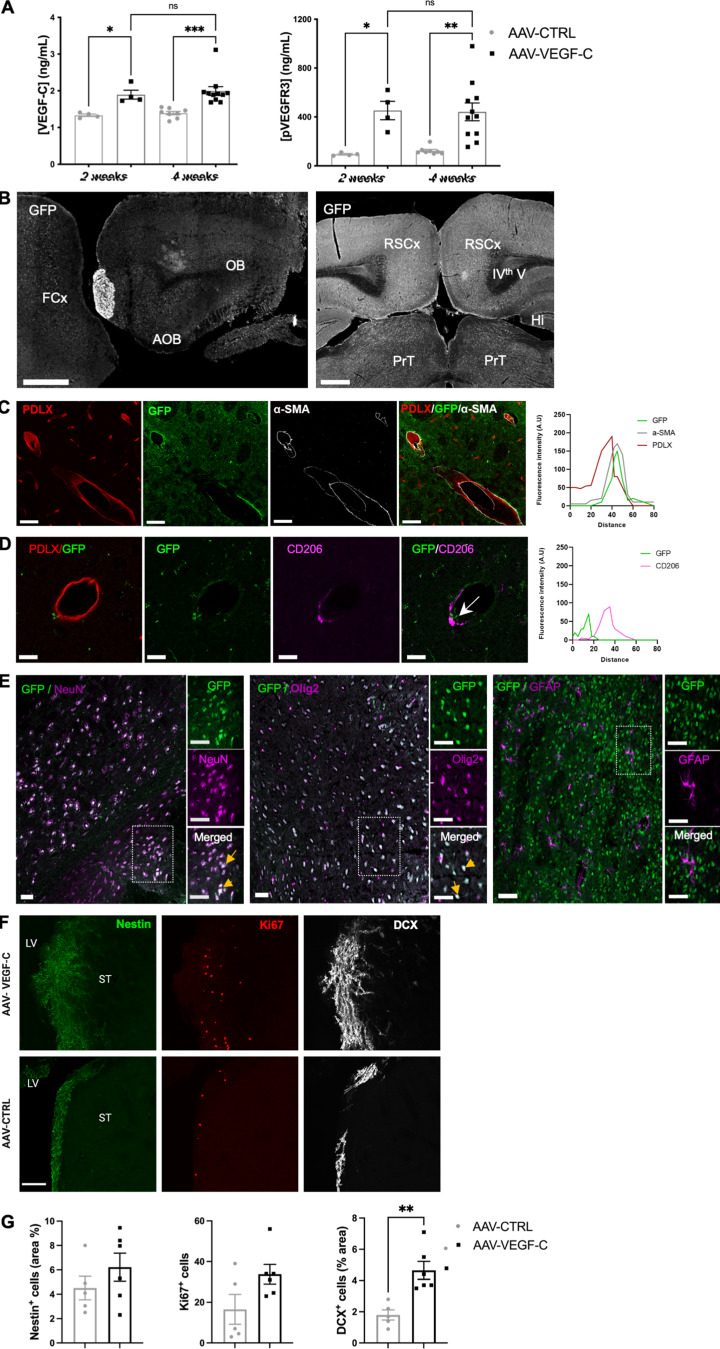
**ICM AAV delivery transduced cells and brain cell responses. (A)** Quantification of VEGF-C and p-VEGFR-3 levels in brain sample lysates using ELISA. ***P < 0.001; **P < 0.005; *P < 0.05 *n* = 4 mice/group at 2 wk *n* = 8–11/group at 4 wk. **(B–E)** Phenotype of brain cells transduced after ICM injection of AAV9-GFP. **(B)** Tile scans of GFP expression on sagittal (left panel) and coronal (right panel) sections of the adult brain. Note the intracerebral GFP expression at the level of the olfactory bulb (OB), accessory olfactory bulb (AOB), frontal cortex (FCx), retrosplenial cortex (RSCx), hippocampus (Hi), and pretectum (PrT). IVth V: fourth ventricle. **(C and D)** GFP is detected in SMA^+^ vascular smooth muscle cells (C), but not in PDLX^+^ endothelial cells and CD206^+^ perivascular macrophages (D). Right panel in C and D: Representative intensity profile plots for GFP (green), a-SMA (gray), PDLX (red), and CD206 (purple), taken from a cross section (white arrow) of the images shown in C and D. Note the co-localization or exclusion of GFP expression with a-SMA and PDLX/CD206 markers, respectively. **(E)** Neural GFP expression is detected in subsets of NeuN^+^ neurons, Olig2^+^ oligodendroglial cells, and very few GFAP^+^ astrocytes (E). Scale bar: 500 μm (B); B–D: 50 μm (C–E). **(F)** Representative images of subventricular zone (SVZ) Nestin^+^ neural stem/progenitor cells, KI6^7+^ dividing cells and DCX^+^ neuroblasts. LV: lateral ventricle; ST: striatum. **(G)** Quantification of Nestin^+^ cells (% SVZ area: AAV-VEGF-C: 6.2 ± 0.9; AAV-control: 4.5 ± 1.1, P = 0.4), KI67^+^ cells (number of cells/surface unit: AAV-VEGF-C: 33 ± 4; AAV-control: 16 ± 7, P = 0.07) and DCX^+^ neuroblasts (% SVZ area: AAV-VEGF-C: 4.6 ± 0.5; AAV-control: 1.7 ± 0.3 **P = 0.004) at 7 days after AAV administration. Data shown as mean ± SEM, *n* = 5–6 mice/group. Scale bar: 100 μm (F).

To test activity and distribution of AAVs following ICM injection, we confirmed by ELISA on brain extracts that AAV-mVEGF-C increased the level of both VEGF-C and phosphorylated (p-) VEGFR-3, thereby enhancing VEGF-C–VEGFR-3 signaling in the brain (Fig. S3 A). Next, we used a control AAV serotype 9-GFP vector and immunophenotyped the fluorescent GFP^+^ AAV_9_-infected cells in the brain at 4 wk after injection (Fig. S3 B). GFP was expressed by perivascular smooth muscle cells but not endothelial cells (Fig. S3 C). Surprisingly, neither pial nor perivascular CD206^+^ macrophages were transduced by AAV_9_-GFP (Fig. S3 D), while subsets of NeuN^+^ neurons, Olig2^+^ oligodendroglial cells, and GFAP^+^ astrocytes expressed GFP (Fig. S3 E).

We tested whether ICM injection of AAV-mVEGF-C could trigger VEGFR-3–mediated neurogenesis (Calvo et al., 2011; Han et al., 2015). Using immunolabeling with anti-doublecortin (DCX) antibodies to detect newborn neuroblasts at 7 days after ICM injection, we found that the subventricular zone of lateral ventricles harbored more DCX^+^ cells in VEGF-C–pretreated mice than in controls (Fig. S3, F and G). This finding indicates that intrathecal injection of AAV-mVEGF-C can stimulate VEGF-C–VEGFR-3 signaling to increase neurogenesis.

**Figure 3.**
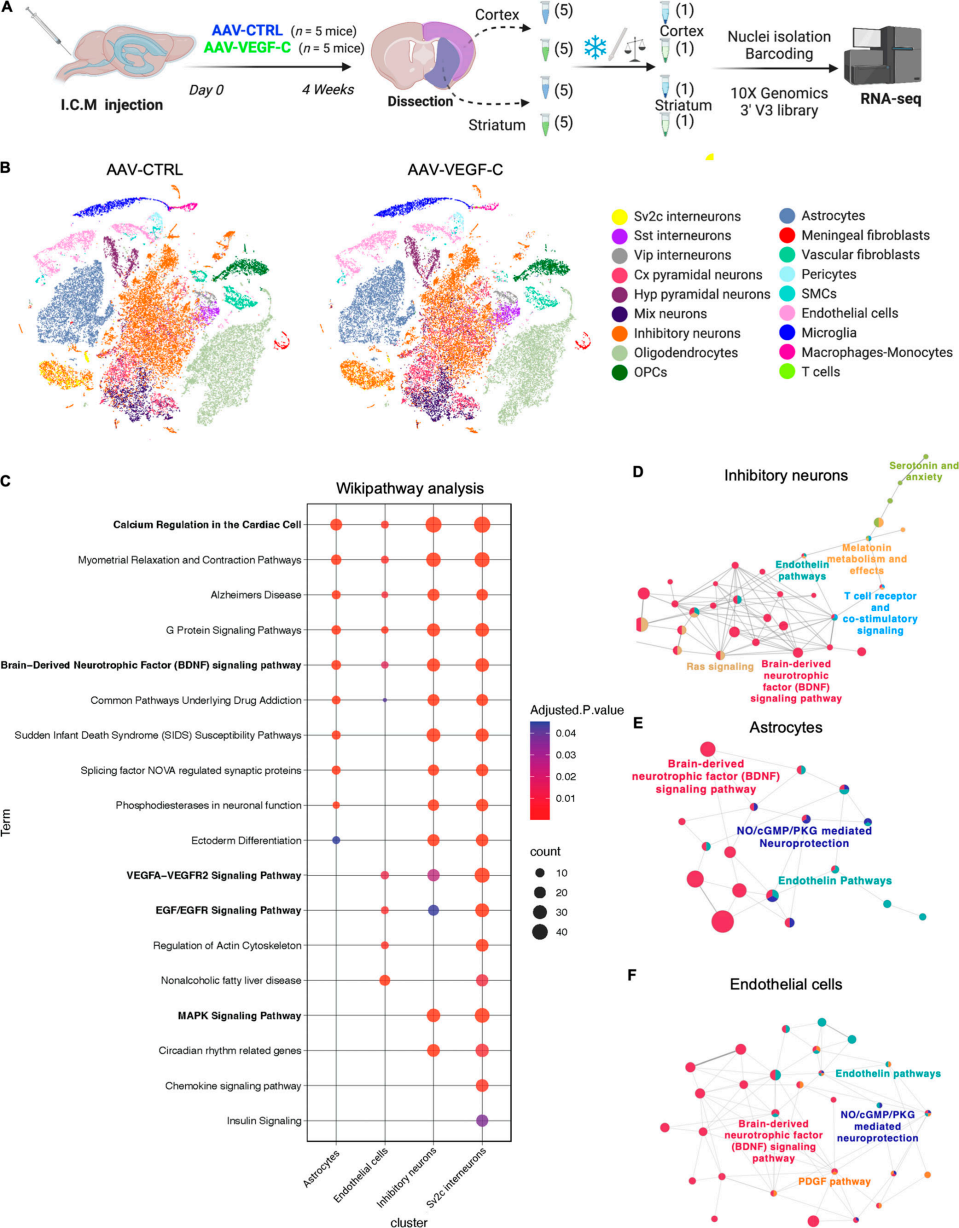
**snRNA-seq reveals VEGF-C induced pathways in forebrain cells. (A)** Overview of the experimental procedure. **(B)** TSNE plots showing cell-type-specific cluster annotation. **(C)** WikiPathways analysis of signaling pathway alterations induced by VEGF-C among brain cell clusters. Oligodendrocytes, astrocytes, endothelial cells, cortical pyramidal (cx pyram) neurons, inhibitory neurons, *Sv2c* interneurons, and mix neurons show >180 DEGs (Padj < 0.05 and abs(log_2_FC) > 0.58), with pathways of at least 20 genes and adjusted P values <0.05 in at least one cluster. **(D–F)** Functionally organized network from ClueGO analysis visualized with Cytoscape in inhibitory neurons (D), astrocytes (E), and endothelial cells (F). Only main pathways are represented. Main terms are represented with color. Dot size represents the number of DEGs in common between pathways. Vip, vasoactive intestinal polypeptide; Hyp, hypothalamus; OPC, oligodendrocyte precursor cell.

**Figure S4.**
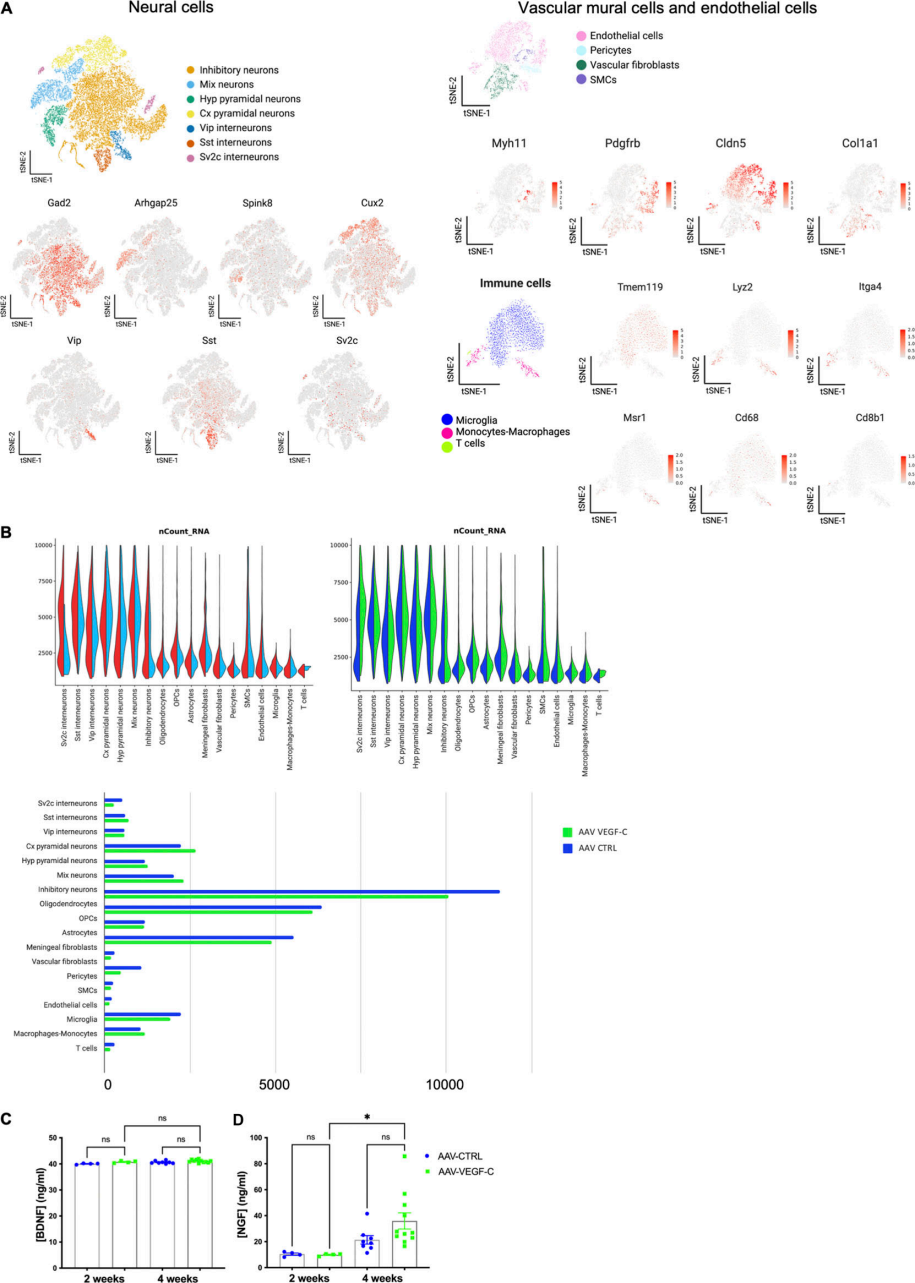
**Single nucleus analysis of brain cell transcriptome in adult AAV-CTRL and AAV-VEGF-C mice. (A)** Subclustering of forebrain neuronal cells, endothelial cells, vascular mural cells, and immune cells. Top panel: tSNE representation of the neurons after sub-clustering and isolated mapping. tSNE representation of marker gene expression in the neuronal cells subclusters. Middle panel: tSNE representation of vascular mural cells and endothelial cells clusters and distribution of relevant marker genes in tSNE representations. Endothelial cells (Cldn5^+^), smooth muscle cells (SMCs) (Myh11^+^); vascular fibroblast (Col1a1^+^); Pericytes (Pdgfrβ^+^ Myh11^−^). Lower panel: tSNE visualization of subclusters of immune cells. Scaled distribution of marker genes of microglia (Tmem119^+^), monocytes-macrophages (Msr1^+^, CD68^+^), and T cells (Cd8b1^+^). **(B)** Violin plots representing transcript number in each cluster, between regions (cortex versus striatum, top left). Between conditions (AAV-VEGF-C versus AAV-CTRL, top right) (*n* = 5 mice/group). Representative histogram of cell numbers in each cluster are shown in the bottom panel. **(C and D)** Quantification of the levels of BDNF (C) and NGF (D) detected by ELISA in brain sample lysates from 2- to 4-wk-treated mice with AAV-VEGF-C or -CTRL. *P < 0.05, *n* = 4 mice/group at 2 wk, *n* = 8–11/group at 4 wk. One-way ANOVA and Tukey’s multiple comparison test. Data are represented as mean ± SEM.

To characterize the molecular changes induced by VEGF-C pretreatment in brain cells, we used snRNA-seq (Schirmer et al., 2019; Zeisel et al., 2015) on cortical and striatal areas dissected from AAV-mVEGF-C and AAV-CTRL mice (*n* = 5 mice/group) (Fig. 3 A). In total, we sequenced 78,728 nuclei with a median of 1,627 genes and 2,550 transcripts per nucleus (Table S1). 18 main clusters were annotated (Fig. S4, A and B). We found similar numbers of neuronal, glial, and non-neural nuclei between AAV-CTRL– and AAV-mVEGF-C–pretreated mice (Fig. S4 B and Table S1). The tSNE representation showed no major differences in clustering after AAV-mVEGF-C treatment (Fig. 3 B). Alteration of transcriptional expression was, however, detectable in several clusters such as *Sv2c* and inhibitory neurons (Fig. S4 B).

Signaling pathway alterations were investigated with the WikiPathways platform and the Enrichr analysis tool, showing that inhibitory neurons and *Sv2c* interneurons, as well as endothelial cells and astrocytes, displayed a high number of altered expression pathways (*n* > 25) and were the most significantly affected by VEGF-C (Fig. 3 C). Pathways of calcium regulation, G protein signaling, Alzheimer’s disease signaling, and BDNF signaling were upregulated in all four cell types. Activation of the BDNF pathway was higher in inhibitory neurons (*n* = 26; P = 2.7.10^−7^) and *Sv2c*-expressing interneurons (*n* = 29; P = 9.02.10^−6^) than in astrocytes (*n* = 12; P = 4.4.10^−5^) and endothelial cells (*n* = 6; P = 0.02). Neurons shared with endothelial cells the upregulation of tyrosine kinase receptor signaling pathways, including mitogen-activated protein kinase, VEGF, and EGF.

We further performed network analysis with Cytoscape and found that VEGF-C upregulated interaction of BDNF pathway with other signaling networks in these clusters (Fig. 3, D–F). Despite transcriptomic evidence of BDNF signaling alteration, ELISA showed that BDNF protein expression in the brain remained unchanged after AAV-mVEGF-C prophylactic treatment (Fig. S4 C). However, a twofold increase in nerve growth factor (NGF) expression was detected (Fig. S4 D), confirming enhanced neurotrophin gene expression in VEGF-C–treated brains.

### Both vascular and neural cells are cellular targets of VEGF-C in the brain

**Figure S5.**
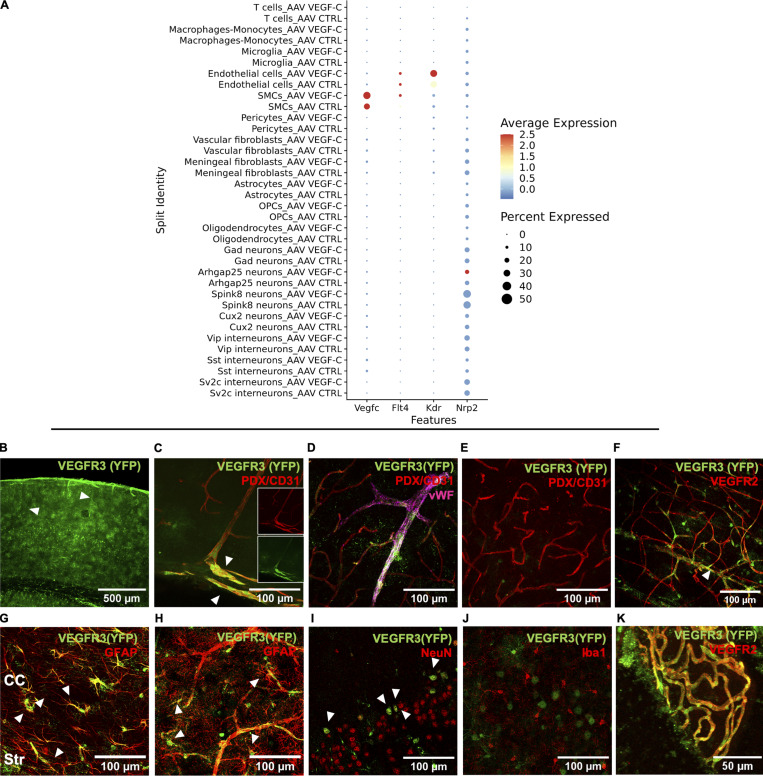
**Analysis of Vegfc and VEGF-C receptor gene expression. (A)** sn-RNAseq analysis. Dot plot representation of transcript expression among the different clusters using Log normalized and zero centered expression in each cluster (AAV-VEGF-C, AAV-CTRL). **(B–K)** Immunophenotyping of *Vegfr3*-expressing cells in the brain of *Vegfr3::YFP* reporter mice. **(B)** Pial and brain penetrating blood vessels (arrowheads) as well as subsets of neural cells express YFP in the cortex. **(C)** Colocalization of YFP and PDLX/CD31 in pial vessels (white arrowhead). **(D)** YFP labeling along large veins expressing Von Willebrand factor (vWF) and exiting the cortex. **(E and F)** Most small cerebral vessels lack YFP expression (E), although YFP can be detected at branching points of VEGFR2^+^ capillaries (F). **(G–K)** Other types of brain parenchymal cells express YFP, including astrocytes and their end-feet (GFAP^+^; white arrowheads in G and H) and subsets of neurons (NeuN^+^; white arrowheads in I). Microglial cells do not express YFP (J), in contrast with VEGFR-2^+^ choroid plexus blood vessels (K).

To identify the subsets of brain cells that may secrete or respond to VEGF-C, we first analyzed the distribution of the transcripts of *vegfc* and VEGF-C receptors’ genes among the cell clusters (Fig. S5 A). *Vegfc* expression was detected in smooth muscle cells and upregulated in these cells by AAV-mVEGF-C administration, in agreement with published data (Antila et al., 2017) and our observation that smooth muscle cells are transduced by AAV_9_-GFP (Fig. S3 B). Smooth muscle cells of the brain perivascular spaces may therefore become an additional source of VEGF-C after intrathecal delivery of AAV-VEGF-C.

Among the cell clusters expressing VEGF-C receptors, we found that endothelial cells expressed little *Flt4* (*vegfr3*) and upregulated *Kdr* (*vegfr2*) upon VEGF-C stimulation. Neuropilin2 (*Nrp2*), coding for a coreceptor of VEGFR-3 (Xu et al., 2010), was detected in neuronal cells including *Sv2c*-expressing interneurons, somatostatin (*Sst*) interneurons, parvalbumin (*Pvalb*) interneurons, and pyramidal neurons, as well as in meningeal stromal cells. To reinforce transcriptomic data, we further investigated brain expression of VEGFR-3 by IHC, taking advantage of *Vegfr3::YFP* reporter mice to detect low levels of *Vegfr3* expression (Calvo et al., 2011). Yellow fluorescent protein (YFP) expression was mainly observed in radial cerebral vessels and astrocytes (Fig. S5 B). YFP labeled large cerebral veins (PDX^+^/CD31^+^, von Willebrand Factor^+^) and subsets of capillaries that also expressed VEGFR-2 (Fig. S5, C–F). YFP was detected in subsets of GFAP^+^ astrocytes (Fig. S5, G and H), as well as in subpopulations of NeuN^+^ neurons, although not in ionized calcium-binding adaptor protein-1 (Iba1^+^) microglia/macrophages (Fig. S5, I and J). Of note, YFP was coexpressed with VEGFR-2 by choroid plexus cells (Fig. S5 K). VEGFR-2 and *Vegf3* are thus coexpressed by subsets of endothelial cells and choroid plexus cells, and *Vegfr3* by subpopulations of neural cells, but not by microglial cells.

### VEGF-C pretreatment ameliorates ischemic stroke outcomes

**Figure 4.**
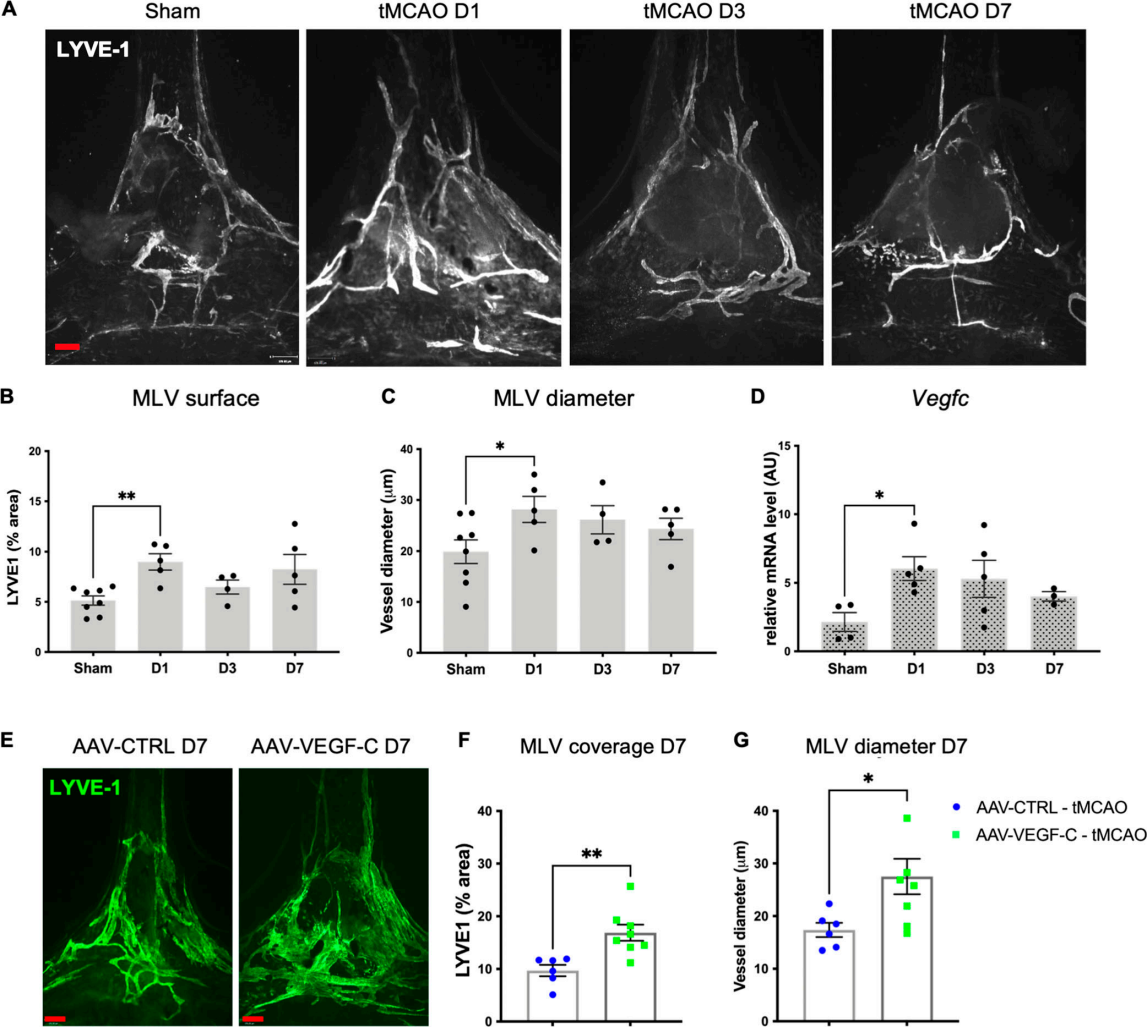
**MLV response to stroke without or with AAV-VEGF-C prophylaxis. (A–D)** Characterization of tMCAO mice. **(A)** Images of LYVE-1^+^ MLVs at the COS in sham mice and mice at 1 d-, 3 d-, and 7 d-pso. **(B and C)** Quantification of the surface (B) and diameter (C) of MLVs at the different time points after stroke compared to the sham group (*n* = 4–8 mice/group, **P < 0.005, *P < 0.05 Mann-Whitney test). **(D)** Quantification by qPCR of *Vegfc* expression on the right hemisphere (forebrain) (*n* = 3–5 mice/group, *P < 0.05 Mann–Whitney test). **(E–G)** AAV-VEGF-C– or AAV-CTRL–treated tMCAO mice. **(E)** Confocal imaging of MLVs labeled with the indicated antibody in the COS. **(F and G)** Quantification of LYVE-1^+^ area (G) and diameter (H) at 7 d-pso (*n* = 6–8 mice/group, *P < 0.05, **P < 0.005; Mann–Whitney). Scale bar: 170 μm (A and E).

We next investigated the direct effects of stroke on MLVs and brain tissues. tMCAO led to a transient expansion of LYVE-1^+^ MLV surface area and diameter in the dorsal dura mater at 1 day–poststroke onset (1 d-pso) that was not sustained at 3 d- and 7 d-pso (Fig. 4, A–C). *Vegfc* transcript expression in the ipsilateral hemisphere likewise transiently increased at 1 d-pso (Fig. 4 D). By contrast, when tMCAO was performed 4 wk after administration of AAV-mVEGF-C or AAV-CTRL, a sustained increase of the LYVE-1^+^ MLV coverage and diameter was induced in VEGF-C–pretreated mice compared to AAV-CTRL mice at 7 d-pso (Fig. 4, E–G).

**Figure 5.**
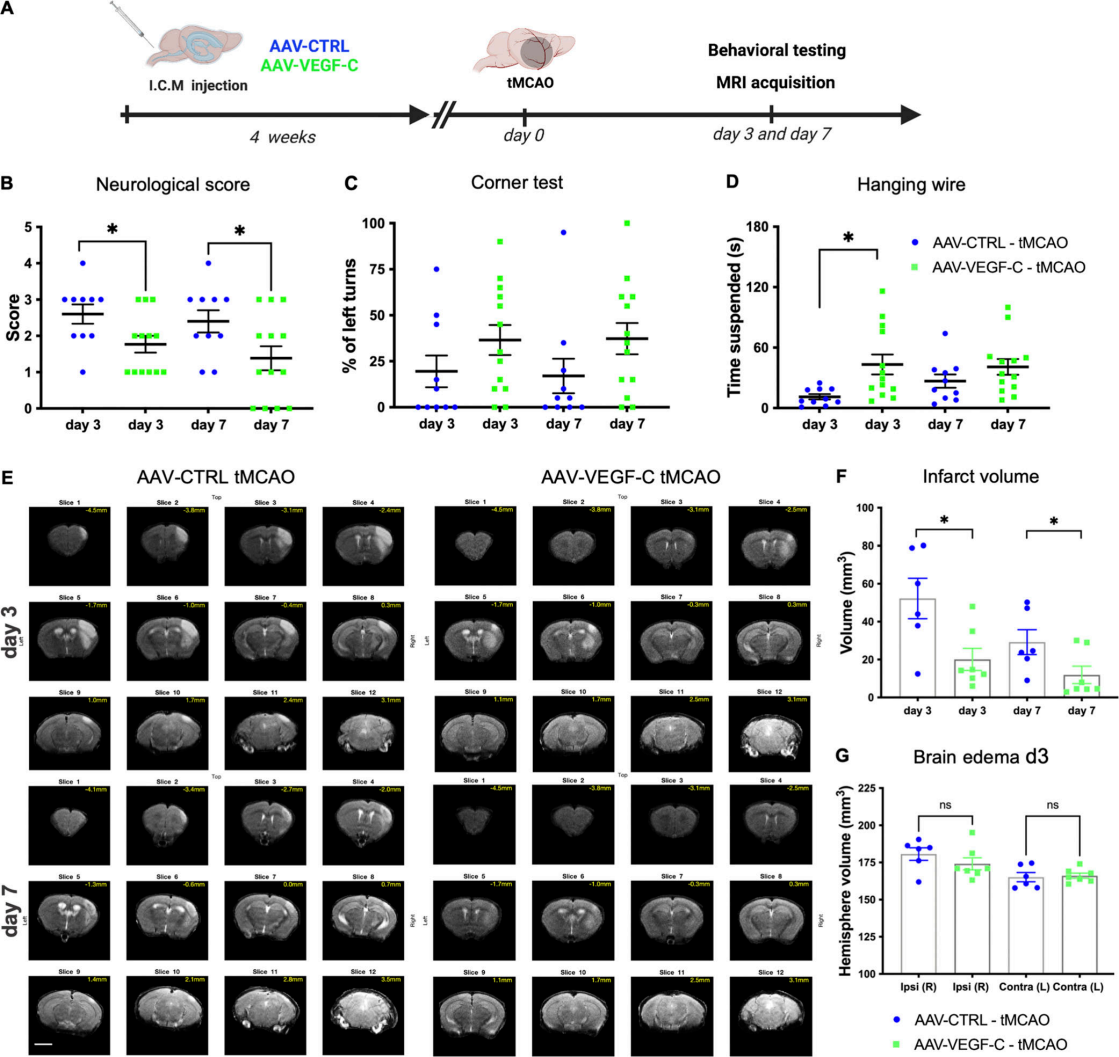
**AAV-mVEGF-C promotes functional recovery in response to stroke. (A)** Overview of the experimental procedure: mice received an ICM injection of AAV-VEGF-C or AAV-CTRL and underwent tMCAO 4 wk afterward. Animals were evaluated using MRI and behavioral tests at 3 d- and 7 d-pso. **(B–D)** Functional analysis of tMCAO mice injected with AAV-VEGF-C and AAV-CTRL. Quantification was performed at the indicated time points. Quantifications of the neurological score (B) and corner test evaluation (C) at 3 d- and 7 d-pso. No difference (% percentage) of left turns (impaired side) between AAV-VEGF-C (day 3: 36 ± 8; day 7: 37 ± 8) and AAV-CTRL (day 3: 19 ± 8; day 7: 17 ± 9. *n* = 10–13 mice/group; P = 0.21). Hanging wire test (D) (*n* = 10–13 mice/group; *P < 0.05; two-way ANOVA followed by Bonferroni’s multiple comparisons). **(E–G)** MRI scans. **(E)** Representative images of MRI anatomical T2 weighted scans showing the infarct lesion induced by tMCAO in AAV-CTRL and AAV-mVEGF-C mice. **(F)** Quantification of the lesion volume at 3 d- and 7 d-pso. (*P < 0.05; Wilcoxon test). **(G)** Volumetric quantification of the ipsilateral and contralateral cerebral hemispheres. (*n* = 6–7 animals/group; Wilcoxon test). MRI scale bar: 400 µm.

tMCAO mice were then subjected to a series of behavioral tests (Fig. 5 A). Neurological outcomes were improved in VEGF-C–pretreated mice compared with controls. The Neuroscore of VEGF-C–pretreated mice was improved at 3 d- and 7 d-pso (Fig. 5 B). Moreover, VEGF-C–pretreated mice showed a similar sensorimotor performance as measured by the corner test (Fig. 5 C). However, at 3 d-pso, the average time of VEGF-C–pretreated mice in the hanging wire test was also longer compared with controls (Fig. 5 D), which reflects less motor neuromuscular impairment, better muscular strength, and motor coordination at the subacute stage.

In vivo MRI of tMCAO mice was used to localize and measure the volume of infarct lesions, corresponding to T2 hyperintense regions (Fig. 5 E). At 3 d- and 7 d-pso, the infarct lesions were significantly reduced in the VEGF-C–pretreated compared with control tMCAO mice (Fig. 5 F). Measurement of edema in the ipsilateral hemisphere of VEGF-C showed no reduction at 3 d-pso when compared with control tMCAO mice (Fig. 5 G). MLV expansion in VEGF-C–pretreated mice thus correlated with the improved sensory-motor behavior and the reduction of nervous tissue damage observed after stroke.

**Figure 6.**
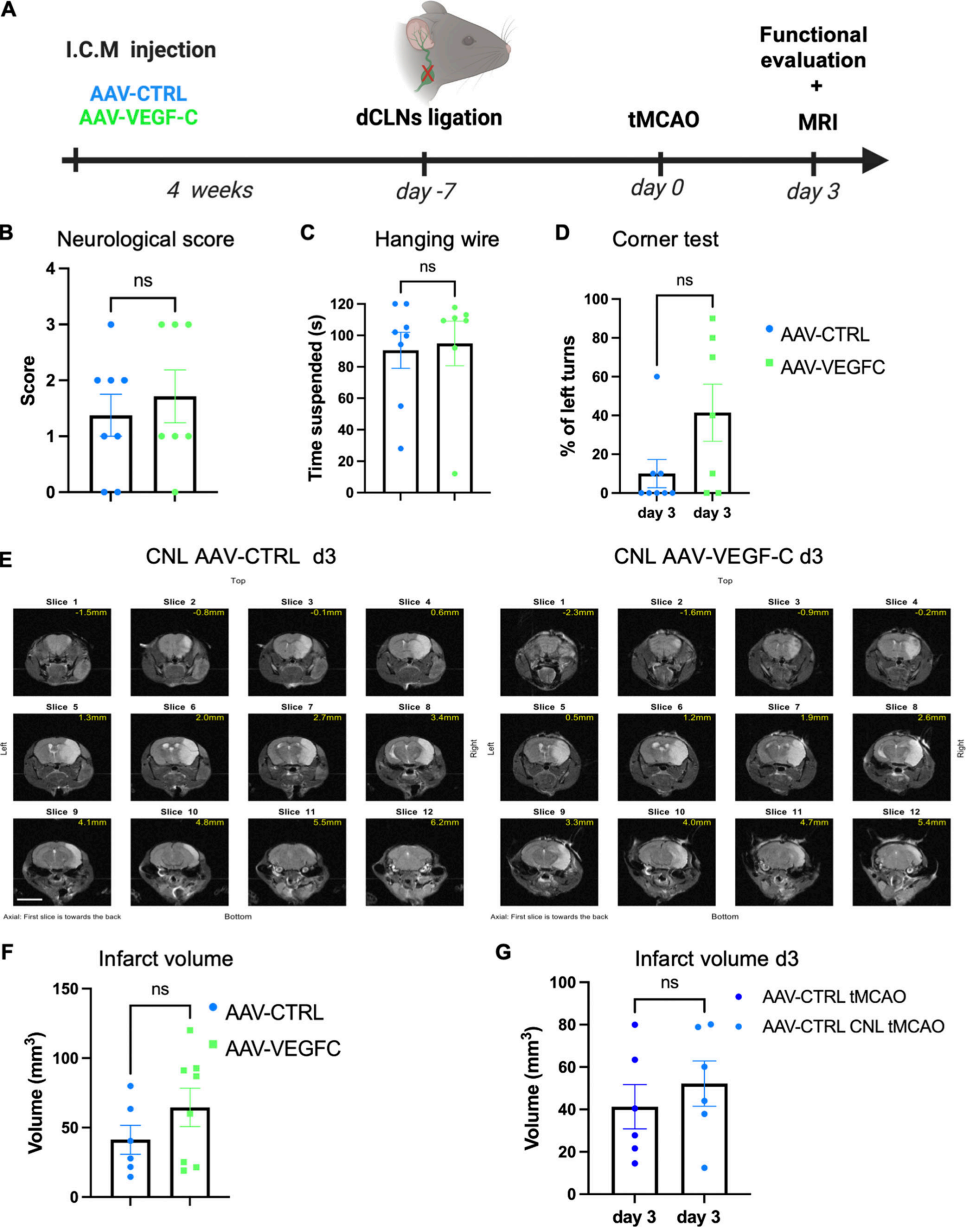
**Post-stroke outcomes after cauterization of dCLN afferent lymphatics. (A)** Mice received an ICM injection of AAV-VEGF-C or AAV-CTRL and 3 wk after underwent CLN ligation; 1 wk later mice were subject to a tMCAO. Animals were evaluated using MRI and behavioral tests at day 3 after stroke. **(B–D)** Functional analysis of tMCAO mice injected with AAV-VEGF-C and AAV-CTRL after CLN ligation. Quantification of the neurological score (B) and hanging wire test (C) and corner test (D) at 3 d-pso (*n* = 7–8 mice/group, ns = no significant difference Mann-Whitney test). **(E)** Representative images of MRI anatomical T2 weighted scans showing the infarct lesion induced by tMCAO in CNL AAV-CTRL and CNL AAV-VEGF-C mice. **(F)** MRI quantification of the infarct volume (*n* = 6–8 animals/group; Mann-Whitney test). **(G)** Comparison between AAV-CTRL mice without and with CLN ligation. Lesion volume measured using MRI at 3 d-pso. Two independent experiments, *n* = 6 mice/group. Scale bar: 500 µm.

To determine whether lymphatic drainage was required for improved stroke outcomes after VEGF-C pretreatment, we cauterized the lymphatic dCLN afferents to block the exit of lymphatic CSF drainage before tMCAO (Fig. 6 A) and examined mice at 3 d-pso. The benefit of AAV-mVEGF-C treatment was lost upon dCLN cauterization: the behavioral performances (Fig. 6, B–D) and the size of cerebral lesions (Fig. 6, E and F) were similar between cauterized AAV-CTRL– and AAV-mVEGF-C–treated mice.

When comparing two independent experiments (AAV-CTRL from Fig. 5 F and Fig. 6 F), we can demonstrate that the cauterization of dCLNs did not change the infarct outcomes of stroke in AAV-CTRL–treated mice (Fig. 6 G). Hence, CSF draining lymphatics mediate, at least in part, the beneficial effect of VEGF-C on the outcomes of tMCAO.

### VEGF-C pretreatment prevents microglia activation and increases neuroprotective factors in the ischemic brain

**Figure 7.**
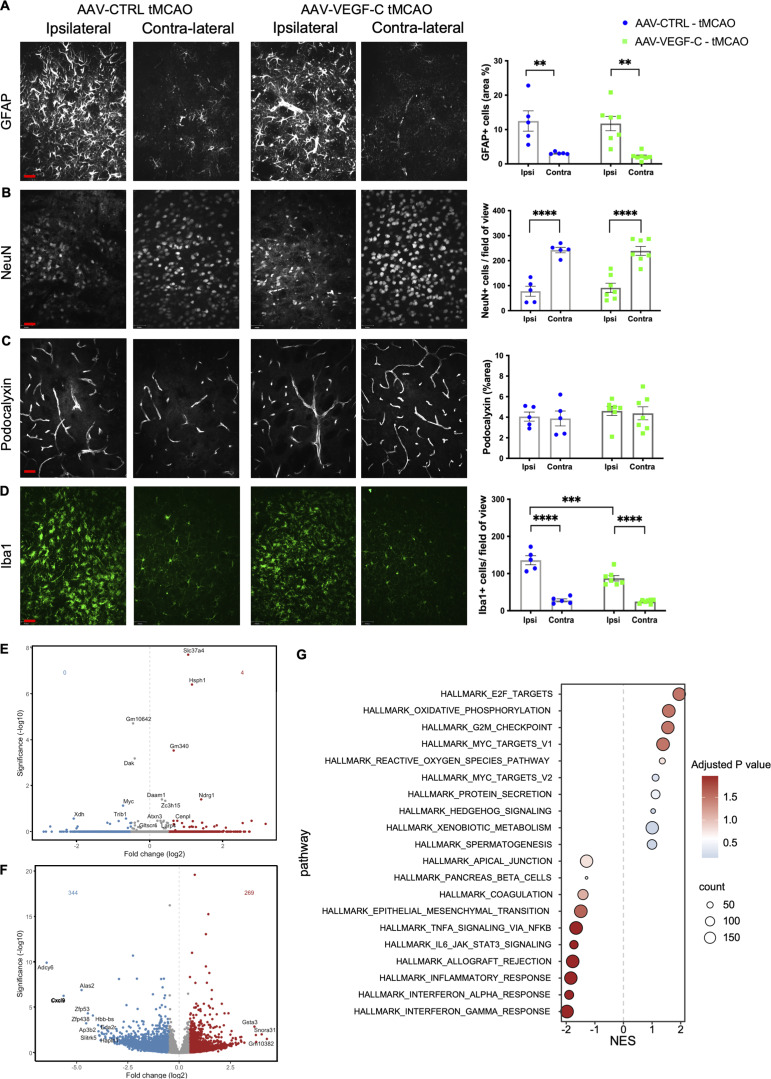
**VEGF-C prophylaxis prevents microglia expansion after ischemic stroke. (A–D)** Representative images and quantification of GFAP^+^ astrocytes (A), NeuN^+^ neurons (B), PDLX^+^ blood vessels (C), and Iba1^+^ immune cells (D) in the ipsilateral (Ipsi) and contralateral (Contra) hemispheres of AAV-CTRL and AAV-VEGF-C mice at 7 d-pso. **P < 0.01, ***P < 0.001, ****P < 0.0001; one-way ANOVA and Bonferroni’s post hoc test. Data are represented as mean ± SEM, *n* = 5–7 mice in each experimental group. **(E–G)** Bulk transcriptomic analysis of CD11b^+^ cells harvested from the brains of mice treated with AAV-mVEGF-C or AAV-CTRL at 7 d-pso or in healthy adults. **(E)** Volcano plot of DEGs between VEGF-C– and CTRL-treated adult mice. **(F)** Volcano plot of DEGs between VEGF-C– and CTRL-treated mice at day 7 after stroke. **(G)** HALLMARK GSEA dot plot showing the most upregulated and downregulated pathways in the AAV-mVEGF-C group compared to AAV-CTRL at 7 d-pso. Scale bar: 35 µm (A–D).

To investigate the histological and gene expression changes induced by VEGF-C pretreatment in tMCAO mice, we performed immunolabeling on 7 d-pso brain sections. We found similar coverage of astrogliosis (GFAP^+^ area), density of perilesional NeuN^+^ neurons, and podocalyxin^+^ blood vessels (PDLX^+^ area) in AAV-mVEGF-C and AAV-CTRL tMCAO mice (Fig. 7, A–C). However, the number of Iba1^+^ microglia and macrophages in the perilesional area was reduced by VEGF-C pretreatment (Fig. 7 D), indicating that VEGF-C may mitigate the inflammatory responses induced by stroke.

To assess the inflammatory response of microglia, we analyzed the transcriptome of CD11b^+^ cells isolated from the brain of AAV-mVEGF-C– and AAV-CTRL–treated in steady-state adult mice (Fig. 7 E), as well as at day 7 after tMCAO (Fig. 7 F). Volcano plots show that the number of DEGs induced by VEGF-C in microglia was strongly increased in mice with stroke compared with mice at steady state. HALLMARK enrichment analysis revealed that microglia inflammatory responses in tMCAO mice were repressed by VEGF-C pretreatment (Fig. 7 G). Pathways related to microglia inflammatory response such as TNF-α/NF-κB, IFN-α, and IFN-γ signaling pathways were inhibited, while oxidative phosphorylation, a marker of non-inflammatory microglia (Orihuela et al., 2016), was increased. Noteworthy, E2F target signaling and DNA replication, but not mitosis, were upregulated by VEGF-C in microglia. Altogether, these molecular data provide evidence that VEGF-C represses inflammatory and proliferative behavior in microglia at 7 d-pso.

**Figure 8.**
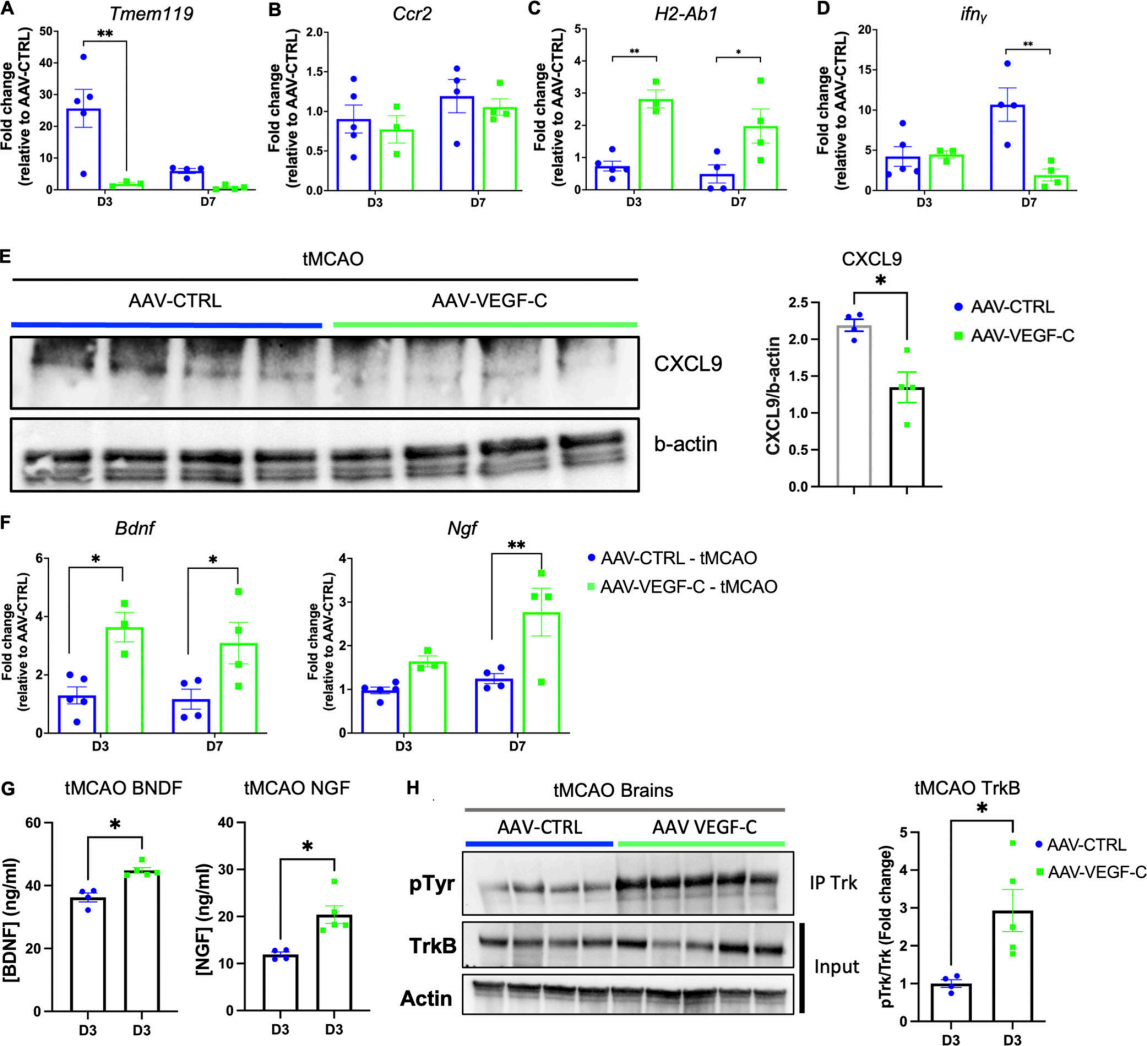
**VEGF-C prophylaxis mitigates microglia activation and stimulates neurotrophin signaling. (A–D)** qPCR analysis of forebrain homogenates (ipsilateral hemisphere) in AAV-VEGF-C and AAV-CTRL mice at 3 d- and 7 d-pso, measuring mRNA expression of the indicated genes. **P < 0.001; *P < 0.05. Two-way ANOVA and Sidak’s multiple comparison test. Data are represented as mean ± SEM, *n* = 3–5 mice in each experimental group. **(E)** Western blot and quantification of CXCL9 protein levels in brain protein extracts from AAV-CTRL– and AAV-VEGF-C–injected mice. *P < 0.05; *n* = 4 animals/group; Mann-Whitney test. **(F)** qPCR analysis of forebrain homogenates described in A–D for measuring mRNA expression of *Bdnf* and *Ngf*. *P < 0.05, **P < 0.01. **(G)** ELISA on brain tissue extracts to measure the expression of BDNF and NGF in tMCAO mouse brains at 3 d-pso (*P < 0.05, *n* = 4–5 animals/group; Mann–Whitney test). **(H)** Immunoprecipitation of TrkB, followed by western blot detection of phosphorylated tyrosine, and quantification of pTrK phosphorylation. Each lane corresponds to one brain hemisphere (*P <0.05; *n* = 4–5 animals/group; Mann-Whitney test). Source data are available for this figure: SourceData F8.

To identify molecular signals that may account for the VEGF-C–induced histological and behavioral changes after stroke, we analyzed brain extracts from control and VEGF-C–pretreated tMCAO mice sacrificed at 3 d- and 7 d-pso. qPCR showed that *Tmem119* expression was strongly reduced by VEGF-C pretreatment compared with control mice (FC = 10 at 3 d-pso), while *Ccr2* expression by macrophages/monocytes was similar between groups (Fig. 8, A and B). VEGF-C increased MHC-II expression (Fig. 8 C), but reduced expression of IFN-γ (*Ifng*), a major proinflammatory cytokine gene induced by stroke in VEGF-C–treated tMCAO mice (Fig. 8 D). Among the top VEGF-C–downregulated microglial genes (Fig. 7 F), we found another proinflammatory chemokine gene encoding the CXCL9 protein (Romagnani et al., 2004), whose expression was reduced by VEGF-C pretreatment (Fig. 8 E).

qPCR of brain extracts showed that VEGF-C pretreatment was associated with a persistent increase in the transcription of neuroprotective *Bdnf* and *Ngf* genes (Fig. 8 F). At 7 d-pso, the expression of BDNF and NGF proteins in the brain of VEGF-C–treated tMCAO mice was upregulated (Fig. 8 G), and phosphorylation of TrkB, the high-affinity receptor of BDNF, was increased (Fig. 8 H). Neuroprotective BDNF/TrKB signaling and NGF expression are therefore promoted by VEGF-C in brain cells after ischemic injury. Altogether, these results demonstrate a dual and beneficial effect of VEGF-C pretreatment for brain tissue after stroke injury by reducing microglia inflammatory pathways and improving neurotrophic signaling.

### Acute VEGF-C treatment does not improve outcomes after ischemic injury

**Figure 9.**
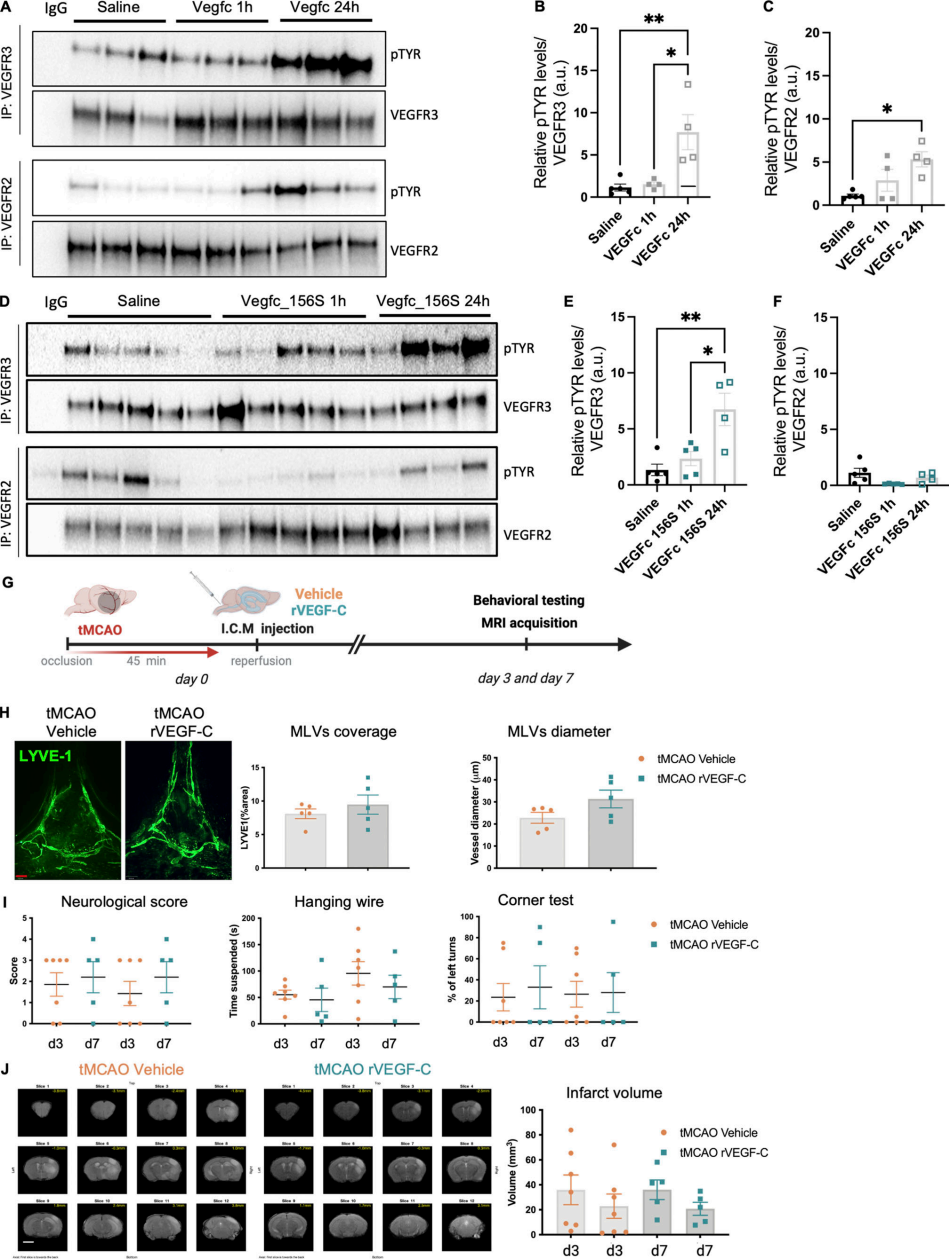
**Single-dose VEGF-C treatment does not improve tMCAO outcomes. (A)** VEGFR-3 and VEGFR-2 immunoprecipitation (IP) of brain protein extracts followed by western blot detection of phosphorylated tyrosine (pTYR) from mice injected with saline or VEGF-C protein (1 μg) at 1 and 24 h after administration (*n* = 3/group). **(B and C)** Quantification of tyrosine phosphorylation levels of VEGFR-3 (B) and VEGFR-2 (C) normalized to immunoprecipitated VEGFR-3 and VEGFR-2 proteins, respectively. *P < 0.05, **P < 0.01. **(D)** VEGFR-3 and VEGFR-2 IP of brain protein extracts followed by western blot detection of phosphorylated tyrosine from mice injected with either saline or VEGF-C156S protein (1 μg) at 1 and 24 h after administration (*n* = 5/group). **(E and F)** Quantification of tyrosine phosphorylation levels of VEGFR-3 (E) and VEGFR-2 (F) normalized to immunoprecipitated VEGFR-3 and VEGFR-2 proteins, respectively. *P < 0.05, **P < 0.01. **(G)** Experimental setting: mice underwent tMCAO and, after reperfusion, received an ICM injection of either recombinant VEGF-C (VEGF-C156S) or vehicle control (0.25% BSA). **(H)** Anti-LYVE-1–immunolabeled MLVs in the COS at 7 d-pso, and quantification of MLV coverage and diameter (*n* = 5 mice/group; Unpaired *t* test). **(I)** Neuroscore scale and corner test quantifications at 3 d- and 7 d-pso (*n* = 7, 5 mice/group; one-way ANOVA test). **(J)** Representative MRI anatomical T2 weighted scans showing the infarct lesion. Quantification of the lesion volume at 3 d- and 7 d-pso (*n* = 7–5 mice/group; Kruskal–Wallis test). Scale bar: 170 µm (H). MRI scale bar: 400 µm. Source data are available for this figure: SourceData F9.

Finally, we tested if the protective effects of VEGF-C could be used as a therapy. 1 μg of recombinant native mouse VEGF-C protein was injected into the CSF and then the brain was collected after either 1 or 24 h. Immunoprecipitation of VEGFR-2 and VEGFR-3 from brain tissue lysates was followed by anti-phospho-tyrosine western blot to assay VEGFR-2/VEGFR-3 phosphorylation. The native form of VEGF-C promoted tyrosine-phosphorylation of both VEGFR-3 and VEGFR-2 in brain cells at 24 h after treatment, but not 1 h after treatment (Fig. 9, A–C). The VEGFR-3–specific VEGF-C156S variant only activated VEGFR-3 with a non-significant trend toward signal increase at 1 h and a significant increase at 24 h (Fig. 9, D–F). Based on its specific and efficient effect on brain VEGFR-3 signaling and to avoid possible blood vessel leakage induced by VEGF-C/VEGFR-2 signaling that may worsen brain damage after stroke, we next chose to deliver recombinant VEGF-C156S (1 μg) into the CSF following tMCAO immediately after reperfusion. The animals were evaluated using the same protocol as for the group treated pre-stroke, including behavioral testing, MRI evaluation, and histological analysis of MLVs (Fig. 9 G). Interestingly, VEGF-C treatment at reperfusion had no effect on MLV coverage (Fig. 9 H). We also observed that VEGF-C156S treatment at this time point had no beneficial effect regarding the functional outcome (Fig. 9 I) or lesion size (Fig. 9 J), suggesting that the effects observed upon AAV-mVEGF-C pretreatment are largely dependent on the expansion of MLVs and the long-term neuroprotective effects of VEGF-C signaling.

## Discussion

The data show that VEGF-C overexpression induced by intrathecal delivery of AAV-mVEGF-C led to a range of molecular responses in endothelium, neural cells, and immune cells in the healthy adult brain and after acute ischemic stroke.

VEGFR-3–expressing LECS are a primary meningeal target of VEGF-C. LECs elicited different types of responses to VEGF-C, which may combine to leverage lymphatic drainage of dCLNs. VEGF-C was found to promote: LEC proliferation in the dCLNs, expansion of dorsal and basal MLVs as well as of extracranial lymphatics collecting into CLNs, and activation of KRAS-DN that may contribute to the increase of MLV diameter by changing the LEC growth response. LECs activated by VEGF-C can moreover mediate secondary effects via secreted chemokines and other secreted factors (Randolph et al., 2017), such as CCL28, a T and B cell homing chemokine (Mohan et al., 2017) that correlated with an enrichment of dural B cells in VEGF-C–treated mice. We also identified a repertoire of secreted osteogenic/osteoclastic factors produced by VEGF-C–stimulated LECs, which may participate in the relationship between the MLVs, the skull, and brain health that was recently documented by Ma et al. (2023).

In the brain, VEGF-C overexpression stimulated neurogenesis and induced transcriptomic responses such as increased calcium signaling in inhibitory neurons and *Sv2c* interneurons as well activation of BDNF pathway across neural cell populations. In addition to stimulating production of new neurons, VEGF-C may thus positively impact inhibitory interneuron activity and synaptic plasticity. It remains to be determined if these effects require VEGF-C receptors, and conditional knockout approaches will be required to determine if they are mediated through VEGFR-3 or other VEGF-C receptors on neurons or if they are indirectly mediated through crosstalk with other VEGF-C responsive cells.

In the context of stroke, we observed an acute but transient increase in MLV coverage and caliber within 24 h. MLV outgrowth after stroke injury also correlated with a surge of cortical *Vegfc* expression, which confirms previous reports of upregulated VEGF-C protein expression in brain cells after ischemia, either within 24 h after focal photo-thrombosis (Jiang and Liao, 2010) or 72 h after 5 min ischemia (Bhuiyan et al., 2015). VEGF-C produced by brain tissues surrounding the lesion may thus trigger a growth response in MLVs, but this response is insufficient to maintain lymphatic expansion and for reducing tissue damage.

Ischemic stroke mice with VEGF-C prophylaxis showed decreased microglial/macrophage proinflammatory gene expression and increased BDNF signaling, associated with better outcomes with respect to lesion size and neurological tests at 3 d- and 7 d-pso. Repression of microglia response translated into a reduction of the Iba1^+^ cell population and *Tmem119* expression associated with a decrease in proinflammatory cytokine production. In contrast, we found that the expression of Ccr2, a chemokine receptor expressed by circulating monocytes/macrophages (She et al., 2022), was not altered in mice with VEGF-C prophylaxis. VEGF-C may thus repress microglia proliferation without increasing infiltration of circulating macrophages, thus limiting post-ischemic inflammation. Depletion of resident microglia in the early stage of stroke has been reported to reduce cerebral ischemic damage (Li et al., 2021). Immunosuppression of microglia may thus contribute to the beneficial effect of VEGF-C prophylaxis in the acute phase after stroke.

Bulk RNA-seq data analysis showed that VEGF-C prophylaxis dampened microglia activation via the downregulation of inflammatory TNF-α, IFN-α, and IFN-γ responses and promoted microglial homeostasis by triggering oxidative phosphorylation. In ischemic stroke, activated microglia play a pivotal role in the initial immune (Ma et al., 2017b) -producing proinflammatory cytokines such as TNF-α and nitric oxide that ultimately contribute to the release of free radicals and mitochondrial damage (Lambertsen et al., 2009). Microglia are unlikely to respond directly to VEGF-C as we could not detect *Vegfr3* expression in these cells. Da Mesquita et al. (2021) have also noted the low frequency of Flt4-expression among microglial cells, and the same authors have shown that impairment of meningeal lymphatic drainage could exacerbate microglial inflammatory response in a model of Alzheimer’s disease. The immunosuppressive effect of prophylactic VEGF-C on microglia observed in mice with ischemic stroke may thus be indirectly mediated by MLVs or cerebral blood vessels expressing VEGFR-3.

Interestingly, VEGF-C is known to have anti-inflammatory effects in a variety of disease models, including inflammatory bowel disease, rheumatoid arthritis, skin inflammation, and wound healing (Schwager and Detmar, 2019). In the skin, stimulation of lymphangiogenesis via VEGFR-3 reduces edema formation by promoting lymph flow (Huggenberger et al., 2010), which in turn promotes immune cell trafficking and triggers the inflammatory response in the injured brain tissues. LECs can directly regulate immune cells and suppress dendritic cell maturation via downregulating expression of proinflammatory markers MHCII, CD40, and IL-6, and increasing expression of anti-inflammatory IL-10 and CCL2 (Christiansen et al., 2016). Conversely, mouse and human microglial activation have been linked with MLV dysfunction as microgliosis was exacerbated by ablation of MLVs in 5xFAD mice, a murine model of Alzheimer’s disease (Da Mesquita et al., 2021). Using gene-set analysis, these authors could correlate microglial activation and impaired MLVs in patients with Alzheimer’s disease. Our present results align well with this study, suggesting that the mitigating effect of VEGF-C prophylaxis on microglia activation may be indirectly mediated by MLV-dependent lymph flow in response to ischemic stroke.

The effect of VEGF-C to promote BDNF signaling noted in the healthy adult mouse brain was amplified after ischemic stroke. Our finding provides evidence to substantiate the hypothesis of BDNF involvement in VEGFR-3–induced neuroprotection, which had previously been proposed about the regulation of dendritic arborization and sensitization of sympathetic vasoconstrictor neurons (Chakraborty et al., 2021). The sustained increase in BDNF signaling may provide additional neuroprotection against ischemic damage, as BDNF is known to promote brain neurogenesis and enhance long-term functional neurological outcomes after cerebral ischemia (Schäbitz et al., 2007). The phenotype of NGF and BDNF-expressing cells activated by VEGF-C and the mechanisms of how VEGF-C activates these cells remain to be characterized.

The immunosuppression of microglial inflammation and the increased neuroprotection induced by VEGF-C pretreatment could not functionally compensate for the loss of CSF lymphatic drainage as the benefit of VEGF-C prophylaxis was lost in mice with cauterized dCLNs. This suggests that the prevalent effect of VEGF-C prophylaxis is to improve lymphatic drainage. In mice with stroke, VEGF-C pretreatment may improve the clearance of brain lesion–derived fluids, debris, and toxins, which in turn decreases meningeal and brain tissue inflammation. In support of this model, K14-Vegfr3-Ig transgenic mice with reduced lymphatic vasculature displayed larger brain lesions than controls after ischemic stroke (Yanev et al., 2020). It is worth noting that an alternative procedure of brain lymphatic blockage by CLN ablation reported the reduction of brain tissue damage and improved motor functions after tMCAO (Esposito et al., 2019; Anthony et al., 2022).

From the perspective of a potential therapeutic approach, we also performed a series of post-stroke treatment experiments using ICM injection of VEGF-C156S. No significant differences in behavioral outcome or lesion size were detected between control- and VEGF-C–treated stroke mice. It is worth noting that VEGF-C156S failed to rapidly induce lymphangiogenesis and promote lymphatic drainage over the analyzed period, which likely precluded a benefit for the stroke outcomes. Recent experiments using direct delivery of VEGF-C156S protein into the carotid artery after tMCAO resulted in larger brain infarcts at both 3 d- and 7 d-pso, compared with control-treated tMCAO mice, without an associated increase in lymphatic growth but with increased local infiltration of CD45^+^ and leukocytes in brain perivascular regions reported (Choi et al., 2023, *Preprint*). Compared with prophylactic ICM administration, VEGF-C delivery into the blood may trigger a broader inflammatory response, thereby worsening brain damage observed after stroke. Collectively these data argue against the use of acute VEGF-C treatment for ischemic stroke. It is possible that VEGF-C administration after stroke activates dCLN immune cells, thereby increasing damage after cerebral ischemia. This would align with our own studies in mice bearing glioblastoma tumors, where VEGF-C rapidly promoted dCLN immune cell activation in response to brain tumor antigens (Song et al., 2020).

To conclude, different, even opposite, outcomes can result from VEGF-C delivery depending upon its timing of administration and the physiopathological context. Here, long-lasting VEGF-C prophylaxis induced by ICM administration before brain injury allowed us to expand brain-draining lymphatics, increase brain waste drainage, and limit brain cell damage by reducing microglial inflammation and increasing neuroprotection. This new information suggests that higher VEGF-C levels in the CSF and augmentation of dural lymphatic drainage activity in individuals may be associated with better outcomes after ischemic stroke.

## Materials and methods

### Study approval and mice

All in vivo procedures used in this study complied with USA federal guidelines and the institutional policies of the Yale School of Medicine Animal Care and Use Committee. Male C57BL/6J mice (purchased from Jackson Laboratory) and *Vegfr3::YFP* mice (Calvo et al., 2011) aged 4–10 wk old were used in the present study.

### Procedures

#### ICM injections

For ICM injections, mice were anesthetized by intraperitoneal (i.p) injection of ketamine (80 mg/kg) and xylazine mix (20 mg/kg). The dorsal neck was shaved and cleaned using povidone–iodine scrub. After positioning the mouse in the stereotactic apparatus, a 1-cm incision was made at the base of the skull, and the dorsal neck muscles were separated using forceps. OVA-A^647^ (Ovalbumin Alexa Fluor 647 Conjugate; O34784; Invitrogen) or AAVs serotype 9 (AAV_9_) were administered by ICM injection. OVA-A^647^ was delivered for either LSFM imaging of CSF drainage (2 mg/ml; 10 μl; 2 μl/min) or quantification of CSF drainage in CLNs (25 mg/ml; 5 μl; 1 μl/min). For AAVs, a single dose of 2 μl (3 × 10^9^ viral particles) of AAV_9_-YFP, AAV_9_ full-length form of mouse VEGF-C (AAV-mVEGF-C) or AAV_9_-mouseVEGFR34–7-Ig (AAV-CTRL) was administered into young (4 wk old) C57BL/6J male mice. For the post-stroke experiment, a single dose of 1 μg of recombinant protein VEGF-C^156S^ was diluted in 2 μl of sterile PBS containing 0.1% BSA (vehicle). ICM injections were performed using a Hamilton syringe with a 34-G needle at a 15° angle. The needle tip was retracted 2 min after the injection. All AAVs were produced by the vectorology platform of the Paris Brain Institute.

#### Focal cerebral ischemia

Focal cerebral ischemia–reperfusion was induced as described previously (Longa et al., 1989). In brief, transient focal ischemia was induced under ketamine (80 mg/kg i.p.) and xylazine (10 mg/kg i.p.) anesthesia. Body temperature was maintained at 37.0 ± 0.5°C throughout the procedure with the help of a homeothermic monitoring system (55-7020; Harvard Apparatus). After a midline neck incision, the right external carotid and pterygopalatine arteries were isolated and cauterized. The internal carotid artery was lifted and occluded at the peripheral site of its bifurcation as soon as the distal common carotid artery was clamped. Focal cerebral ischemia was induced by intraluminal filament occlusion of the right middle cerebral artery (MCA) for 45 min using a 6-0 nylon monofilament with a silicone-coated tip (Doccol Co.). Reduction in regional cerebral blood flow was confirmed using trans-cranial laser-Doppler flowmetry (MoorVMS-LDF1; Moor instruments) in the cerebral cortex supplied by the MCA. Sham-operated mice were anesthetized and the common carotid artery was dissected free from surrounding connective tissue, but the MCA was not occluded. In the present study, a total of 108 animals received tMCAO and the mortality rate was 27.7%. 31 mice died before completing the end of the study and eight animals were excluded for not presenting lesions on MRI or for presenting a hemorrhage.

#### dCLN cauterization

We performed dCLN cauterization according to the procedure described previously (Song et al., 2020). In brief, mice were anesthetized using ketamine and xylazine and the rostral neck was shaved and disinfected. A 2-cm incision was made and the salivary glands containing the sCLNs were retracted and dCLNs were visualized. The afferent LNs were tied off with a 4–0 Vicryl suture and then cauterized. The incision was closed with 4–0 Vicryl suture and mice were subjected to the same post-operative procedures as above.

### Cell and nucleus isolation procedures

#### Flow cytometry isolation of dural lymphatic endothelial and immune cells

Mice were anesthetized by i.p. administration of xylazine and then euthanized with lethal doses of euthasol and transcardially perfused with 20 ml of PBS (*n* = 10–12 for each AAV-treated group). Skullcaps were dissected and harvested using small surgical scissors and put directly in ice-cold PBS. Dural meninges were peeled off the skullcaps using small forceps and transferred to tubes for enzymatic digestion in RPMI containing 2.5 mg/ml collagenase D (11088866001; Roche) + 0.1 mg/ml DNase (11284932001; Roche) for 20 min at 37°C. Following enzymatic digestion, manual pipetting up and down (25 times) was applied, cellular suspensions were passed through a 35-μm pore cell strainer, and cells were transferred to 96 v-well plaques after centrifugation (4 min, ×2,000 *g* at 4°C). The cell preparation from two pooled meninges was collected in each well (150 μl of PBS) for flow-cytometry sorting of dural LECs and CD45^+^ immune cells.

To reduce unspecific antibody binding, cell preparations were first incubated for 10 min at 4°C with anti-CD16/32 (FC block; 5 μg/ml; BD Biosciences). This step was followed by adding the fluorescent conjugated antibodies cocktail: Live-or-Dye^405/452^ (32003; 0.05 μl/test; Biotium), CD31 PerCP-Cy5.5 (102420; 1 μl/test; BioLegend), CD45 Viogreen (130-123-900; 1 μl/test; Miltenyi), Podoplanin-PE (12-5381-82; 1 μl/test; eBiosciences), and Lyve-1 Janelia Fluor 549 (FAB2125I; 1 μl/test; Biotechne). Incubation with antibodies lasted 20 min at 4°C and was followed by centrifugation (5 min, × 500 *g* at 4°C) and a single PBS wash. Cells were finally suspended in 600 μl of PBS and sorted using the MoFlo Astrios. Dural LECs were characterized as viable singlets positive for CD31, and Podoplanin and Lyve-1 samples were stored in 1.5-ml tubes filled with TRIzol reagent at −80°C.

#### Magnetic-activated cell sorting of brain microglia/macrophage cells

For the isolation of microglia and infiltrating macrophages, mice submitted or not to MCAO after ICM treatment with either AAV-CTRL or AAV-mVEGF-C were intracardially perfused with ice-cold PBS to remove circulating leukocytes and brains were dissected. Brains were then dissociated with the Neural Tissue Dissociation kit (P) (Miltenyi Biotec) according to the manufacturer’s instructions. The digestion was stopped by adding BSA (5% final) and the cells were immediately filtered through a 70-mm cell strainer. After centrifugation (600 *g* for 5 min), cells were resuspended in PBS supplemented with 2 mM EDTA and 0.5% BSA. Myelin was removed using Myelin Removal Beads II (Miltenyi Biotec), and microglia and infiltrating macrophages were isolated using CD11b MicroBeads (Miltenyi Biotec) according to the manufacturer’s instructions. After bead selection, cells were centrifuged (×800 *g*) for 5 min at 4°C and the cell pellets were frozen at −80°C on DNA/RNA Shield (Zymo research).

#### Brain nuclei isolation

To generate single nuclei transcriptome profiles for mouse brains, we used a total of 10 brain samples from adult male mice (8 wk old). Five brains from AAV-mVEGF-C and AAV-CTRL mice were collected, cryopreserved, pooled, and then processed according to the procedure of nuclei isolation, as previously reported (Ma et al., 2022).

### RNA extraction, sequencing of libraries, and data analysis

#### snRNA-seq

Microfluidic capturing of nuclei and libraries was prepared according to the manufacturer’s protocol (Chromium Single Cell 3ʹ GEM, Library & Gel Bead Kit v3, 16 rxns PN-100007; 10x Genomics). The sequencing was performed by following the manufacturer’s direction (CG000183; 10x Genomics) for targeted 30,000 reads per nucleus on HiSeq 4,000 platform (Illumina). MEX files obtained were then loaded into Seurat, cells presenting <200UMI were discarded, and the samples were normalized using the SCTransform function with regression of the mitochondrial RNA percentage. Batch effects were corrected using the Seurat integration pipeline (Butler et al., 2018) on the 5,000 most variable genes. We then aggregated transcriptionally similar cells using the Seurat t-distributed stochastic neighbor embeddings (tSNE) algorithm. Next, we removed clusters of low quality resulting from debris, doublets/multiplets, and dead cells by regressing out the clusters expressing <1,000 counts on average.

The 78,728 nuclei remaining after processing were clustered using the FindCluster function with k-nearest neighbors of 15 and a resolution of 1.2. The clusters were then manually identified according to their top markers with the help of the Allen Brain atlas (Reference Atlas, 2021). A subclustering of the identified endothelial, microglial/macrophage clusters was then realized using a resolution of 0.8 of the (shared) nearest-neighbor graph construction matrix object with the same default k-nearest neighbors (Waltman and van Eck 2013).

We next conducted differential expression analysis on clusters showing >180 DEGs (P_adj_ < 0.05 et abs(log_2_FC) > 0.58). We used the FindMarkers function from the Seurat pipeline and a Wilcoxon test for P value estimation with a Bonferroni correction for the number of tests. Only differentially upregulated genes (adjusted P value above 1.5) were used for enrichment analysis. The enrichment analysis was performed with EnrichR R packages on the Wikipathways database. We selected pathways with at least 20 genes and with a P value under 0.05. Cytoscape (ClueGO) application was used to visualize enrichment analysis results into functionally organized networks. This approach allowed us to identify the most prominent and statistically significant changes induced by AAV-VEGF-C treatment, although it failed to identify subpopulations of brain cells such as neural stem/progenitor cells and immune cell subtypes, and we could not detect *Vegfr3* transcripts in brain interneurons and astrocytes, despite their expression of YFP reporter gene in *Vegfr3::YFP* brains.

#### Bulk RNA-seq of FACS-isolated dural cells

RNA extractions were performed using the Direct-zol RNA Microprep kit (R2061) according to the manufacturer’s instructions. RNA library preparation was realized following the manufacturer’s recommendations (SMARTer Stranded Total RNA-Seq Kit v3—Pico Input Mammalian from Takara). Final samples pooled library preparations were sequenced on Nextseq 2,000 ILLUMINA with a P2-200 cycles cartridge (2 × 400 millions of 100 base reads) corresponding to 2 × 66 millions of reads per sample after demultiplexing. Differential analysis was done with the DESeq2 method and gene ontology (GO) enrichment using the Gene Set Enrichment Analysis (GSEA) in the Quby-RNA tool of the Data Analysis Core facility at the Paris Brain Institute.

Volcano plots, heatmaps, and bubble plots were generated using the open-source web apps VolcaNoseR (Goedhart and Luijsterburg, 2020), Heatmapper (Babicki et al., 2016), and SRPlot (https://www.bioinformatics.com.cn/en), respectively.

#### Bulk mRNA-seq of magnetic-activated cell sorting–isolated brain CD11b^+^ cells

RNA isolation was performed on frozen cell pellets on DNA/RNA Shield (Zymo Research) using Direct-zol RNA Miniprep kit (Zymo Research). RNA concentration was measured by NanoDrop 1000 (Thermo Fisher Scientific), and RNA integrity was determined using Bioanalyzer (Agilent). Library construction of 300 ng total RNA for each sample was made using KAPA Stranded mRNA-Seq Kit (Illumina Platforms; Kapa Biosystems) using 10 cycles of PCR amplification. Libraries were purified using AxyPrep Mag PCR Clean-up kit (AxygenTM). Each library was quantified using a Qubit fluorometer (Life Technologies) and the size distribution was assessed using the 2,100 Bioanalyzer (Agilent Technologies). Sequencing was performed on an Illumina Hiseq 2,500 (Illumina) instrument using the TruSeq PE Cluster Kit V4-cBot-HS (Illumina) to generate 101-bp paired-end reads sequencing with v4 chemistry. Quality control of RNA-Seq reads was performed using FastQC v0.11.9, samtools v1.7, and Picard v2.23.8. Transcripts were quantified with Salmon v1.4.01 and reads aligned using STAR v2.7.62 to the mouse genome (mm10/GRCm38, gencode vM24). Downstream analysis was performed in R and differential expression was analyzed with DESeq2 v1.30.14 with standard models and normal shrinkage estimators. GSEAs were performed with fgsea v1.16.0 using log_2_FC estimates from DESeq2 and gene sets from MSigDB or internal data, as indicated.

### Quantitative molecular analysis techniques

#### Real-time PCR analysis, western blot, and ELISA

qPCR to quantify expression of *Cncnad1*, *Dcn*, *Tmem119*, *Ccr2*, *Bdnf*, and* Ngf* was performed according to Geraldo et al. (2021).

Western blot to quantify CXCL9, VEGFR-2, VEGFR-3, and TrkB expression, as well as immunoprecipitation and anti-phosphotyrosine blotting experiments to quantify p-VEGFR-2, p-VEGFR3, and pTrkB were carried out, as reported (Boyé et al., 2022).

*ELISA* tests for VEGF-C, BDNF, NGF, and p-VEGFR-3 were performed from CSF or brain extract samples, as reported (Geraldo et al., 2021). The sandwich ELISA method with the DuoSet ELISA Ancillary Reagent Kit 2 (R&D Systems) was used according to the manufacturer’s instructions with a set of antibodies listed in Table S2. HRP-linked anti-sheep secondary antibodies (1:1,000) were used for revelation.

### Collection and quantification of OVA-A^647^ in dCLNs

At the indicated times following the injection, mice were euthanized, and CLNs were carefully dissected and put in 2-ml tubes containing 200 µl of formamide solution (AM9342, AM9344). At this step, the sCLNs were separated from the dCLNs. To ensure that the CLNs were submerged in the liquid, tubes were centrifuged for 5 min at 10,000 *g*. This step was followed by 48 h of incubation at 65°C. After that, an OVA-A^647^ curve was prepared by serial dilutions from the ovalbumin stock solution in formamide (20/10/5/2.5/1.25/0.625/0.3125/0.015625 μg/ml). Extracts were then added to the 96-wells plate (100 μl/well) and readings were performed using the Spectramax i3X plaque reader by Molecular devices.

### Behavioral testing

Three behavioral tests were used to functionally assess the sensorimotor function after tMCAO surgery. The behavioral tests were conducted at 3 d- and 7 d-pso by an evaluator blinded to the experimental conditions. Neurological deficit was evaluated using a five-point scoring system (0, no deficit; 1, forelimb weakness and torso turning to the ipsilateral side when held by the tail; 2, circling to one side; 3, unable to bear weight on affected side; and 4, no spontaneous locomotor activity or barrel rolling) as described previously (Liu et al., 2009).

The hanging wire test evaluates both limb strength and balance after MCAO. The apparatus consists of a 50-cm-wide, 2-mm-thick metallic wire, secured to two vertical stands at around 30 cm above a foam pillow. The time-out period was 180 s for each trial. The average time of suspension in three different trials with 5 min rest was then calculated.

The corner test is commonly used for identifying and quantifying sensorimotor and postural asymmetries (Zhang et al., 2002). The apparatus consists of two acrylic boards placed closely together at a 30° angle forming a narrow alley. A mouse is then placed in between the boards facing the corner. As the animal approaches the corner, both sides of the vibrissae are simultaneously stimulated which leads the animal to rear and turn 180°. Animals with unilateral brain damage will preferentially turn around in the ipsilateral direction (non-impaired side). The percentage of left turns in a total of 20 trials was calculated.

### MRI

#### Contrast agent-labeled CSF MRI for glymphatic–lymphatic transport

All the MRI experiments were conducted at the Magnetic Resonance Research Center at Yale University. T1 mapping was used to evaluate glymphatic transport and brain-derived fluid drainage to the CLNs after VEGF-C administration. MRI acquisitions were performed on a Bruker 9.4 T/16 MRI scanner with a BGA-9S-HP imaging gradient interfaced to a Bruker Advance III console and controlled by Paravision 6.1 software (Bruker Bio Spin). The 3D T1 mapping technique used in this study followed the previously published protocol (Xue et al., 2020).

For the CSF contrast administration and MRI procedures, all mice (*n* = 20, 10 injected with AAV-mVEGF-C and 10 with AAV-CTRL) were anesthetized with a ketamine/xylazine (KX) mixture: (ketamine 17.5 mg/ml and xylazine 2.5 mg/ml, 0.1 ml/20 g body weight) combined with glycopyrrolate (0.2 mg/kg i.p.). Anesthesia was maintained with KX (0.05 ml of the KX-mixture/20 g body weight) administered every 30 min via an i.p. catheter and supplemented with a 1:1 air/O_2_ mixture. CSF administration of Gd-DOTA (Guerbet LLC) was performed via the cisterna magna as previously described (Xue et al., 2020). Briefly, we used a 34-ga needle connected via polyurethane tubing to a 50 µl Hamilton syringe (Hamilton) mounted in a microinfusion pump (Legato 130; KD Scientific, MW 559 Da). Gd-DOTA (7 µl) prepared as a 1:20 dilution in sterile 0.9% NaCl was administered at an infusion rate of 1 µl/min. After the CSF infusion, the anesthetized mouse was transferred to the 9.4T MRI for T1 mapping. The T1 scan was prefixed to start 50 min from initiation of CSF infusion on the bench. T1 maps covering the head and neck of the mouse were acquired in two steps: first, a spatial inhomogeneity profile of the radiofrequency transmit (B1+) was acquired using a double angle method using a rapid acquisition with relaxation enhancement (RARE) sequence (repetition time [TR] = 10,000 ms, time to echo [TE] = 22 ms, Average = 1, RARE factor = 4, number of slices = 36, in-plane resolution = 0.24 mm/pixel, slice-thickness = 0.3 mm, slice gap = 0.2 mm, Flip angles = 70° and 140°). Second, a spoiled gradient echo variable flip angle steady state spoiled gradient recalled echo (SPGR) imaging method (TR = 16 ms, TE = 3 ms, Average = 1, scanning time = 2 min 40 s, matrix = 100 × 100 × 100 reconstructed at 0.18 × 0.18 × 0.18 mm). A set of six flip angles (2°, 5°, 10°, 15°, 20°, and 30°) was used, and the total scan required ∼16 min. The T1 maps were filtered and T1 values >5,000 ms were excluded. MRIs comprising the summed low flip angle (2° and 5°) SPGR images were used as anatomical templates for outlining the brain and lymph nodes. The T_1_ maps of the head were used to quantify the volume of glymphatic transport which was defined as brain tissue voxels with a T1 in the range of 1–1,700 ms (this specific T1 range represents tissue that has been shortened by the uptake of Gd-DOTA). For each mouse, the glymphatic transport volume was extracted using PMOD software (PMOD, version 4.0). Similarly, outflow to the nasal conchae and drainage to the dCLNs were quantified by first outlining these anatomical structures and then extracting voxels with T1 values from 1 to 1,700 ms.

#### Measurement of infarct and edema volume

MRI-based translational imaging was used to determine the infarct volume after tMCAO. A cohort of animals was evaluated at 3 d-pso and again at 7 d-pso. Briefly, MRI data were obtained on a modified 11.7T system with a Bruker spectrometer (Bruker Bio Spin). Mice were anesthetized with 2% isoflurane and maintained in a mixture of 1% isoflurane, 30% O_2_, and 70% N_2_O using a nose cone. Respiration rate (50–80 breaths/min) and rectal temperature (37 ± 1°C) were continuously monitored and maintained using a warm water–pumped system. MR images were acquired using a transmit-only volume (70-mm birdcage) coil and receive-only surface (35-mm-diameter ring) coil configuration (RAPID MR International). First, anatomical images were acquired using a T2-weighted RARE sequence with two averages and a RARE factor of 4 (TE = 24 ms, TR = 3,000 ms, field of view [FOV]: 32 × 32, Matrix: 128 × 128, slice thickness: 1 mm, number of slices = 9). T2 measurements were acquired using a Multi-Slice Multi-Echo sequence (number of echoes = 10; TR: 3,000 ms; TE [ms]: 10, 20, 30, 40, 50, 60, 80, 90, and 100; matrix: 85 × 85; FOV: 17 × 17; slice thickness: 0.7 mm; and number of slices: 12). A custom written script using MATLAB version R2019b (MathWorks) was used to fit the data and generate R2 (i.e., 1/T2) maps from the acquired data. The infarct lesion was defined in a semiautomatic fashion. The anatomical images were used to exclude ventricles and to delimitate the brain tissue using MATLAB. Following, we used BioImage suite software (https://medicine.yale.edu/bioimaging/suite/) to detect hyperintense regions on the T2-map using a threshold. The total stroke volume was calculated as the sum of the voxels included in the hyperintense regions across all slices multiplied by the total slice thickness. To test the effect of treatment on edema formation after MCAO, we measured the volume difference between ipsilateral and contralateral hemispheres between treatments.

### Immunohistology and confocal imaging

#### Tissue preparation

Mice were deeply anesthetized with isoflurane and then perfused transcardially with cold phosphate-buffered saline (PBS), followed by 4% paraformaldehyde (PFA). Head dissection was done as described previously (Antila et al., 2017). The dissected skullcaps with the dura mater were post-fixed overnight, washed in PBS, and processed for staining.

#### Immunolabeling of whole-mount preparations of skullcaps

For whole-mount staining of the meninges, the fixed tissues were blocked with 10% donkey serum, 2% BSA, and 0.5% PBS-Triton-X (blocking solution) overnight. Primary antibodies (Table S2) were diluted in DIM, and the samples were incubated in the primary antibody mix at least overnight at 4°C. After washes with PBS-Triton-X at room temperature, the tissues were incubated with fluorophore-conjugated secondary antibodies in PBS-Triton-X overnight at 4°C, followed by washing in 0.5% PBS-Triton-X at room temperature. After post-fixation in 1% PFA for 5 min, and washing with PBS, the stained samples were transferred to PBS containing 0.02% sodium azide at 4°C and imaged.

For labeling with anti-fibrinogen (1/200), skullcaps were first decalcified by immersion into Surgipath Leica decalcifier solution for 2 h, blocked overnight in blocking solution (0.3% Tween20 and 5% donkey serum in PBS1X buffer), followed by coincubation of primary antibodies with appropriate dilutions in the blocking solution for 3 days and secondary antibodies for 2 days.

#### Immunolabeling of sections of the brain and CLNs

For floating sections of the brain, the brain samples were removed and post-fixed overnight. Sections 30 µm thick were obtained using a vibratome. Sections were incubated in a blocking solution for 2 h at room temperature, followed by overnight incubation with primary antibodies. After washing with 0.3% PBS-Triton-X three times, the sections were incubated with fluorescent dye–conjugated secondary antibodies in PBS with 3% donkey serum for 2 h at room temperature. After three washes with 0.3% PBS-Triton-X, the sections were mounted with a fluorescent mounting medium (Dako) between a glass slide and cover glasses.

For cryosections of CLNs, they were post-fixed overnight in 4% PFA and cryopreserved in 30% sucrose. Sections (20 μm) were obtained using a cryostat (model). For cryosections of the head to detect olfactory bulb surrounding lymphatic vessels, the fixed tissues underwent decalcification with 0.5 M EDTA, pH 7.4, at 4°C for 7 days as described (Antila et al., 2017). Samples were washed with PBS and immersed in PBS containing 20% sucrose for 24 h at 4°C, embedded in OCT compound (Tissue-Tek), and frozen. 30-μm sections were immunostained, as described above.

### Confocal microscopy: Image acquisition and analysis

Laser scanning confocal images of the fluorescently labeled brain and whole-mount skullcap mice were acquired using a spinning-disk confocal (Nikon Eclipse Ti) microscope or a Confocal SP8 X White Light Laser Leica microscope (X4) with Z stack (step size = 5 μm) and maximal intensity projection.

Quantitative analysis of meningeal lymphatic coverage and diameter was performed using FIJI Image-processing software (National Institutes of Health). The percentage of the area covered by LYVE-1^+^ MLVs was detected and quantified semiautomatically in the confluence of sinuses. The diameter of MLVs was calculated by the total area of the vessel/length, as described previously (Zhang et al., 2018). The mean value of eight different segments of the MLVs was then calculated.

For the mouse brain samples, we obtained confocal images of immunolabeled cryosections (20× magnification) in three different areas of interest, namely the ischemic core, the border zone, and the contralateral hemisphere. Three brain coronal sections per mouse (containing dorsolateral striatum) were used for quantification of the density of GFAP^+^, PDLX^+^ cells, and the number of microglial cells (Iba1^+^) and neurons (NeuN^+^) (5–6 sections/brain). The percentage of the labeled area by GFAP^+^ or PDLX^+^ cells was calculated in a semiautomatic fashion using FIJI. The number of Iba1^+^ cells and NeuN^+^ cells was manually counted in the different areas of interest, and then their average values were calculated (*n* = 5–6 sections/brain).

### iDISCO^+^-LSFM imaging

Mice were sacrificed at 30 min after ICM injection of OVA-A^647^ and then perfused with 20 ml of PBS 1X, then 20 ml of 4% PFA in PBS. Animals were beheaded and head samples were post-fixed in 20 ml of 4% PFA in PBS for 24 h. Procedures for skull decalcification, iDISCO^+^ tissue clearing and immunolabeling, LSFM imaging, as well as for LSFM image processing and analysis are detailed in (Jacob et al., 2022). We used LSFM (Ultramicroscope II, LaVision Biotec) equipped with a sCMOS camera (Andor Neo) and a 4×/0.3 objective lens (LaVision Biotec).

### Statistical analyses

All graphs and statistical analyses were produced using GraphPad Prism 8. Results were expressed as mean ± standard error of the mean. Experiments were performed with full blinding, allocation concealment, and randomization. The data normal distribution was evaluated using the Shapiro–Wilk test. Two-group comparisons were analyzed using unpaired t-tests, either Mann-Whitney tests or Wilcoxon analyses, depending on the variable’s characteristics. The effect of two independent variables on a dependent variable was assessed using two-way ANOVA. P < 0.05 was statistically significant.

### Graphical illustrations

Graphical illustrations were made using BioRender (https://biorender.com/).

### Online supplemental material

Fig. S1 shows the effects of AAV-VEGF-C on the dural blood vasculature, the dural and extra-cranial lymphatic vessels, and the CLNs. Fig. S2 shows flow cytometry isolation and bulk RNA-seq analysis of dural LECs and immune cells. Fig. S3 shows the phenotype of brain cells transduced after AAV9-GFP ICM administration and the effects of AAV-VEGF-C on the generation of newborn neurons (DCX^+^ cells). Fig. S4 shows a tSNE representation of snRNA-seq subclustering (related to Fig. 3), transcript numbers by cluster, as well as BDNF and NGF levels at 2 and 4 wk after AAV-VEGF-C administration. Fig. S5 shows the analysis of VEGF-C and VEGF-C receptor gene expression and the immunohistological phenotype of *Vegfr3*-expressing cells. Videos 1 and 2 show LSFM images of the pattern of LYVE-1^+^ lymphatic vessels and OVA-A^647^ CSF tracer in the head of AAV-VEGF-C and AAV-CTRL and treated mice, respectively. Table S1 contains a list of the number of nuclei per cluster. Table S2 presents the list of antibodies used in the study.

## Supplementary Material

Table S1 contains a list of the number of nuclei per cluster.

Table S2 presents the list of antibodies used in the study.

SourceData F8 is the source file for Fig. 8.

SourceData F9 is the source file for Fig. 9.

## Data Availability

Data supporting the findings of this research article are available upon request to the corresponding author. The scRNA-seq data from mouse meningeal samples were deposited in the NCBI Gene Expression Omnibus database accession numbers: GSE252093, GSM7993579, GSM7993580, GSM799358, GSM7993582, GSE252115, GSM7993748, GSM7993749, GSM7993750, GSM7993751, GSM7993752, and GSM7993753. The bulk RNA-seq data from mouse brain CD11b^+^ cells were deposited in the NCBI Gene Expression Omnibus database accession number: GSE252622. The bulk RNA-seq data on the effect of VEGF-C treatment on sorted brain microglia/macrophages was deposited in the NCBI Gene Expression Omnibus database accession numbers: GSM8004097, GSM8004098, GSM8004099, GSM8004100, GSM8004101, GSM8004102, GSM8004103, GSM8004104, GSM8004105, GSM8004106, GSM8004107, and GSM8004108. The snRNA-seq data from mouse brain samples were deposited in the NCBI Gene Expression Omnibus database accession numbers: GSE253051, GSM8012966, GSM8012967, GSM8012968, and GSM8012969.
