# Supplementary material for: VEGF-C prophylaxis favors lymphatic drainage and modulates neuroinflammation in a stroke model
Source: J Exp Med. 2024 Mar 5;221(4):e20221983. doi: 10.1084/jem.20221983 (PMC10913814; doi:10.1084/jem.20221983)
Supplement: Table S1 — contains a list of the number of nuclei per cluster. [file jem_20221983_tables1.docx]

| **Cluster** | **AAV VEGF-C** | **AAV CTRL** |
| --- | --- | --- |
| Pvalb+ Neurons | 422 | 335 |
| Sst+ Neurons | 2250 | 2084 |
| Sv2c+ Neurons | 15135 | 17505 |
| Vip+ Neurons | 684 | 585 |
| Mix Neurons | 3427 | 2837 |
| Pyramidal Neurons | 12319 | 10888 |
| Oligodendrocytes | 9287 | 8494 |
| OPCs | 1493 | 1623 |
| Astrocytes | 8674 | 9340 |
| Ependymal Fibroblasts | 375 | 715 |
| Meningeal stromal cells | 1073 | 1856 |
| Perivascular Fibroblasts | 276 | 556 |
| Pericytes | 322 | 384 |
| SMC | 40 | 86 |
| Endothelial cells | 1747 | 1748 |
| Microglia | 2370 | 1988 |
| Monocytes/Macrophages | 236 | 302 |
| Lymphocytes | 163 | 188 |

**Table 1**
