# Supplementary material for: VEGF-C prophylaxis favors lymphatic drainage and modulates neuroinflammation in a stroke model
Source: J Exp Med. 2024 Mar 5;221(4):e20221983. doi: 10.1084/jem.20221983 (PMC10913814; doi:10.1084/jem.20221983)
Supplement: Table S2 — presents the list of antibodies used in the study. [file jem_20221983_tables2.docx]

**Table 2**

**IHC Primary Antibodies:**

| **Antibody Description** | **Dilution** | **Catalog/Code** | **Supplier** |
| --- | --- | --- | --- |
| Chicken anti-mouse GFP | 1:1000 | GFP-1020 | AVES |
| Goat anti-mouse Podocalyxin | 1:400 | MAB1556 | R&D |
| Goat anti-mouse/rat CD31 | 1:400 | AF3628 | R&D |
| Rabbit anti-mouse Lyve-1 | 1:400 | 11-034 | Angiobio |
| Goat anti-mouse Lyve-1 | 1:250 | AF2125 | R&D |
| Chicken anti-mouse GFAP | 1:500 | GFAP | Aves |
| Goat anti-mouse VEGFR2 (Flk-1) | 1:200 | AF644 | R&D |
| Rabbit anti-Human Von Willebrand Factor (vWF) | 1:400 | A0082 | Dako |
| Rabbit anti-mouse GFAP | 1:400 | GTX108711 | Genetex |
| Rabbit anti-mouse NeuN | 1:200 | GTX133127 | GeneTex |
| Rat anti-mouse CD3e | 1:200 | MAB4841 | R&D |
| Rabbit anti-mouse Ki67 | 1:200 | ab16667 | abcam |
| Rat anti-mouse/human/fish CD45R (B220) | 1:200 | 14-0452-85 | Invitrogen |
| Goat anti-mouse CD45 | 1:250 | AF114 | R&D |
| Chicken anti-mouse Nestin | 1:500 | NB100-1604 | Novusbio |
| Mouse anti-human Doublecortin (E-6) | 1:100 | sc-271390 | Santa Cruz |
| Rabbit anti-mouse Iba1 | 1:400 | W1W019-19741 | Wako |
| Rabbit anti-mouse smooth muscle actin | 1:1000 | 14395-1-AP | ProteinTech |
| Goat anti-mouse CD206 | 1:500 | AF2535 | R&D |
| Rat anti-mouse GFAP | 1:500 | 13-03000 | Invitrogen |
| Rabbit anti-mouse Olig2 | 1:250 | AB9610 | Millipore |
| Rabbit anti-mouse fibrinogen | 1:200 | PA5-21968 | Invitrogen |

**IHC Secondary Antibodies:**

| **Antibody Description** | **Dilution** | **Catalog/Code** | **Supplier** |
| --- | --- | --- | --- |
| Alexa Fluor 488-conjugated donkey anti-chicken | 1:1000 | 703-545-155 | Jackson ImmunoResearch |
| Alexa Fluor 594-conjugated donkey anti-goat | 1:500 | 705-585-147 | Jackson ImmunoResearch |
| Alexa Fluor 647-conjugated donkey anti-rat | 1:500 | 712-605-153 | Jackson ImmunoResearch |
| Alexa Fluor 555-conjugated donkey anti-rabbit | 1:500 | A31572 | Invitrogen |
| Alexa Fluor 568-conjugated donkey anti-rabbit | 1:500 | A10042 | Invitrogen |

**FACS Antibodies:**

| **Antibody Description** | **Supplier** |
| --- | --- |
| Anti-CD16/32 FC block | BD Biosciences |
| Live-or-Dye 405/452 | Biotium 32003 |
| CD31 PerCP-Cy5.5 | Biolegend 102420 |
| CD45 Viogreen | Miltenyi 130-123-900 |
| Podoplanin-PE | eBiosciences 12-5381-82 |
| Lyve-1 Janelia Fluor 549 | Biotechne FAB2125I |
| Ovalbumin, Alexa Fluor 647 conjugate | O34784 ; Invitrogen |
