# Supplementary figures and images for: VEGF-C prophylaxis favors lymphatic drainage and modulates neuroinflammation in a stroke model
Source: J Exp Med. 2024 Mar 5;221(4):e20221983. doi: 10.1084/jem.20221983 (PMC10913814; doi:10.1084/jem.20221983)

Figure 8 E

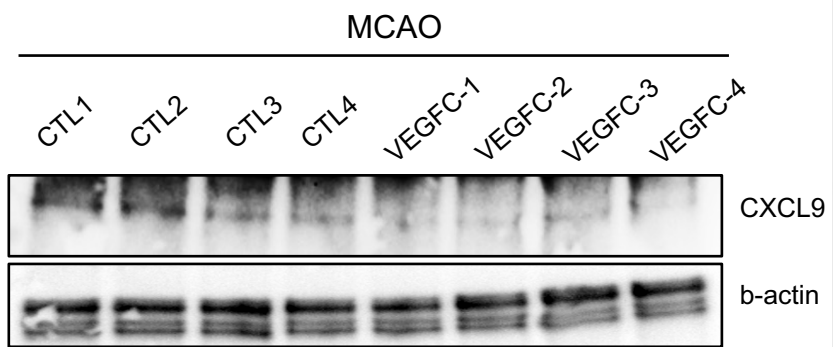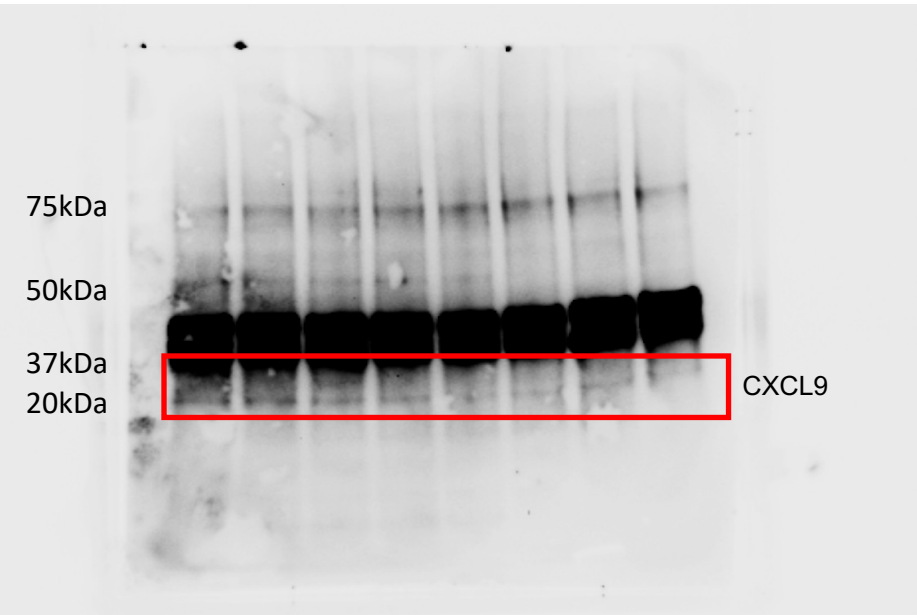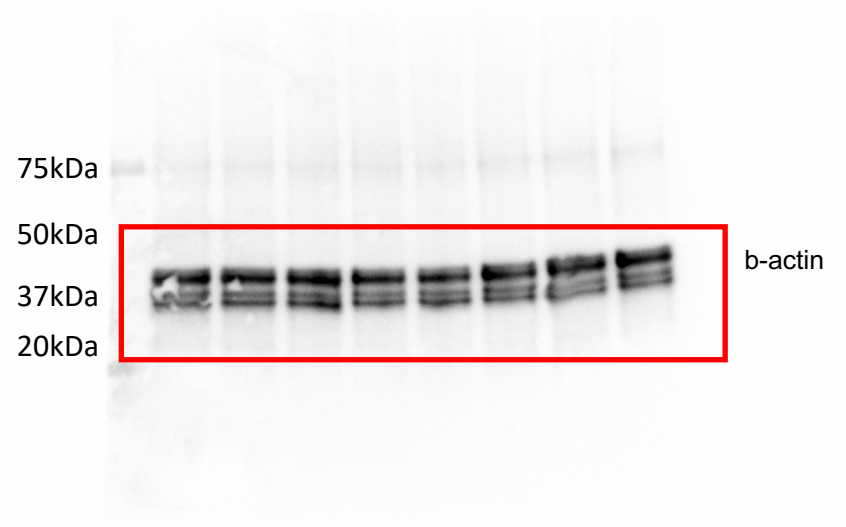

TrkB IP in MCAO Brains – Figure 8H

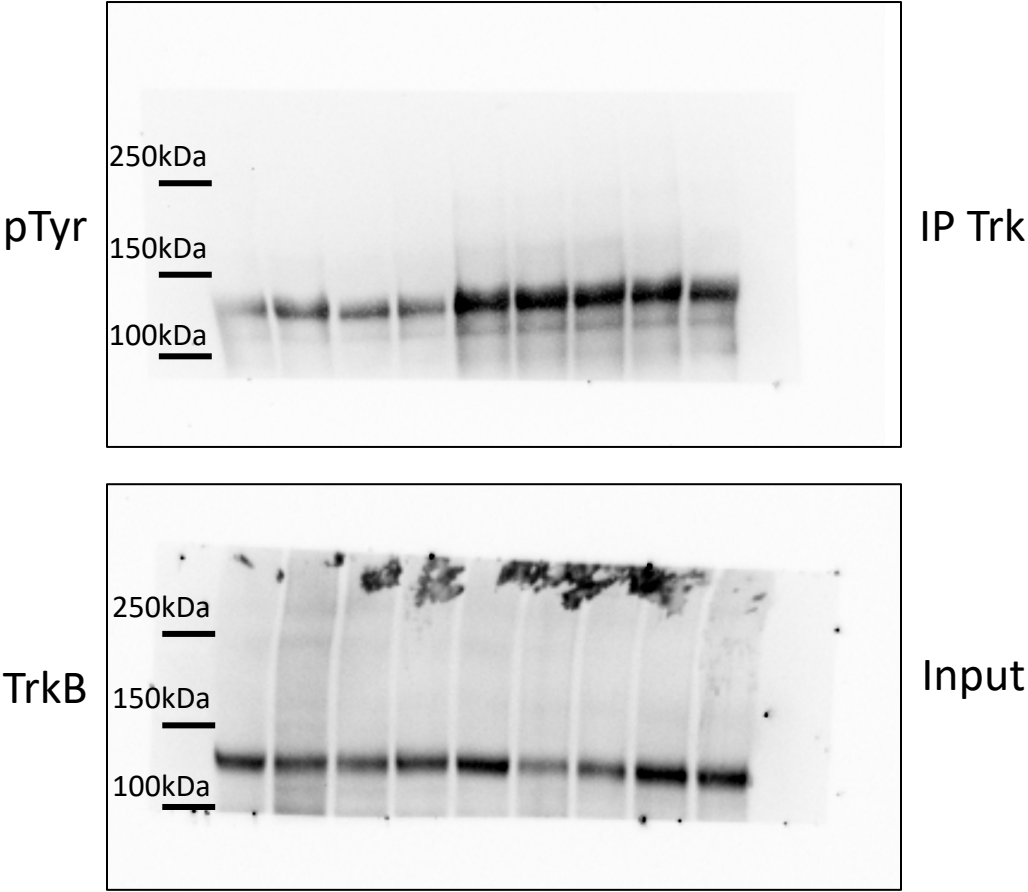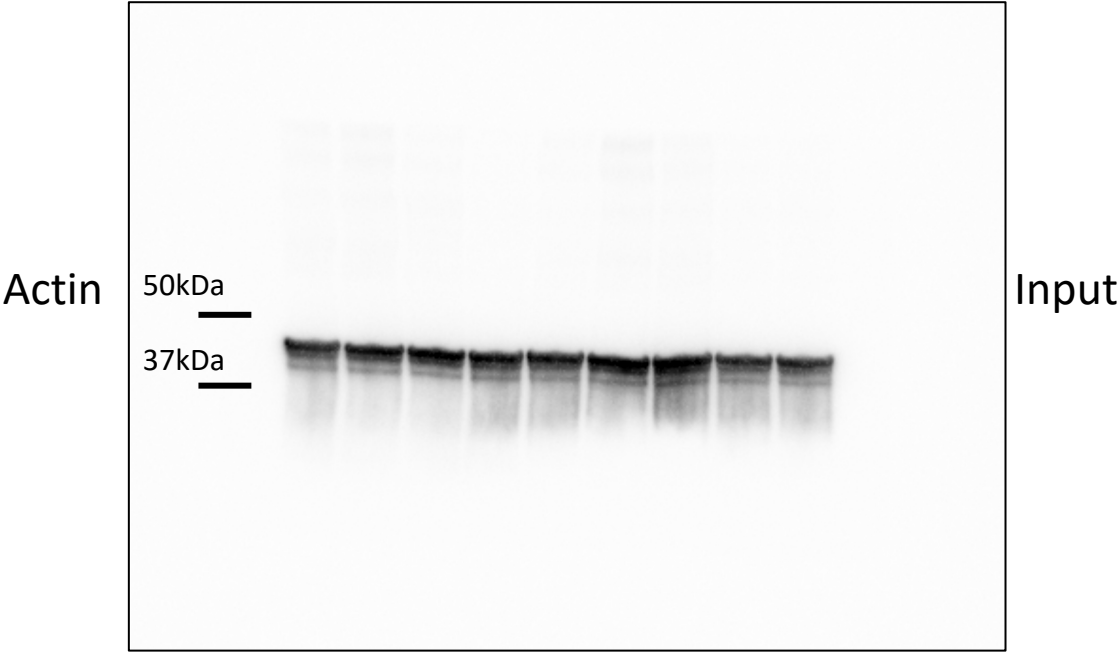

Supplement: SourceData F8 — is the source file for Fig. 8. [file jem_20221983_sourcedataf8.pdf]
