# Supplementary material for: VEGF-C prophylaxis favors lymphatic drainage and modulates neuroinflammation in a stroke model
Source: J Exp Med. 2024 Mar 5;221(4):e20221983. doi: 10.1084/jem.20221983 (PMC10913814; doi:10.1084/jem.20221983)

Figure 9

Vegfc156S_VEGFR3

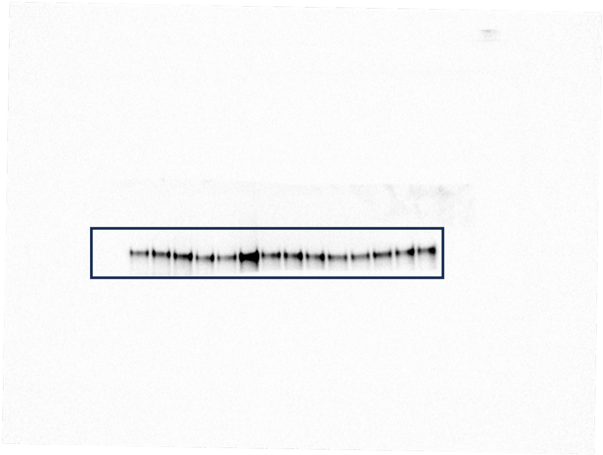

Vegfc156S_VEGFR3_pTYR

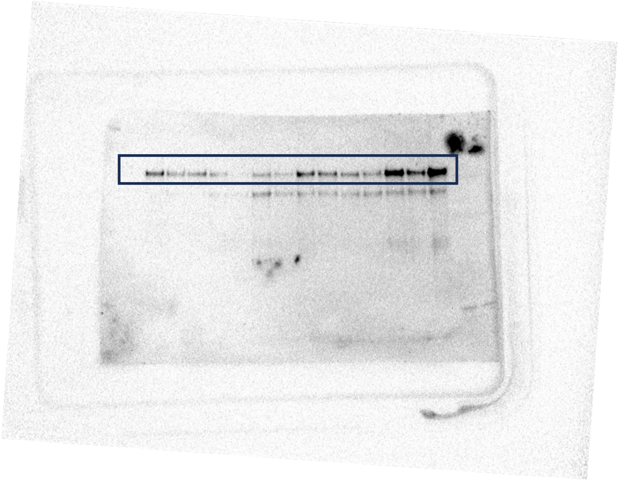

Vegfc156S_VEGFR2

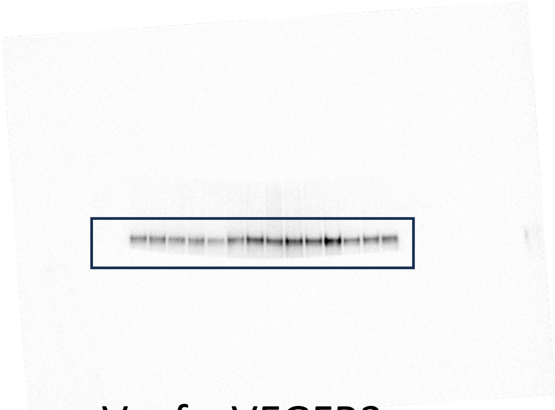

Vegfc156s_VEGFR2_pTYR

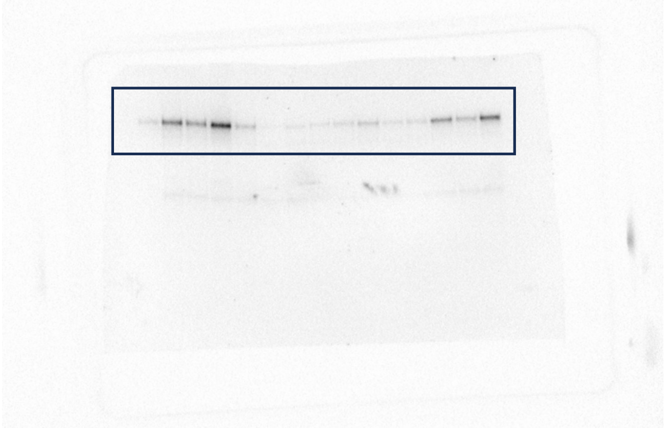

Vegfc_VEGFR3

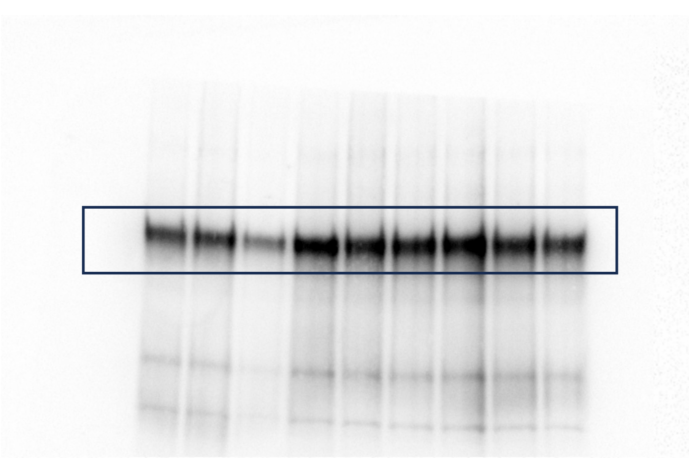

Vegfc_VEGFR2_pTYR

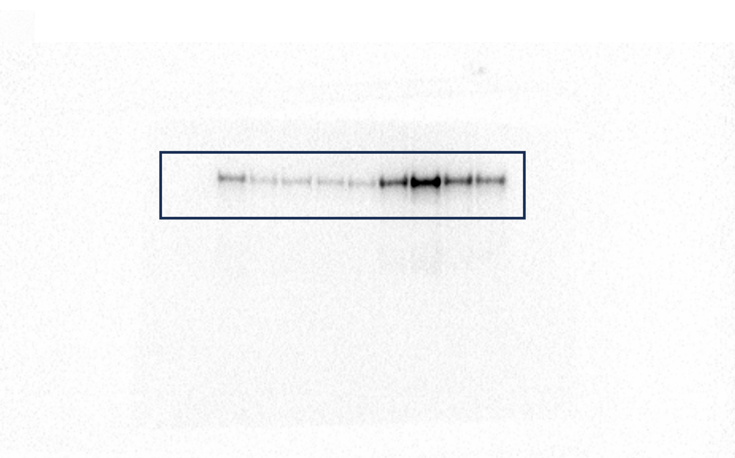

Vegfc_VEGFR2

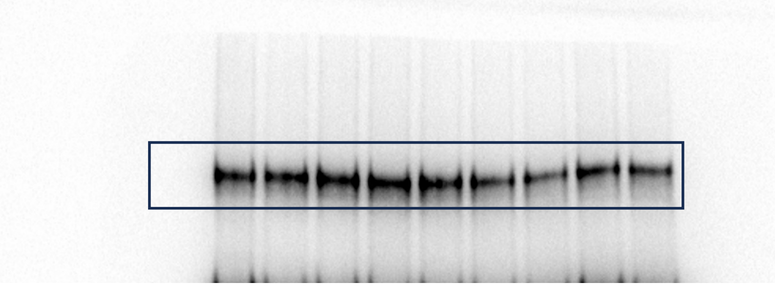

Vegfc_VEGFR3_pTYR_Right to left

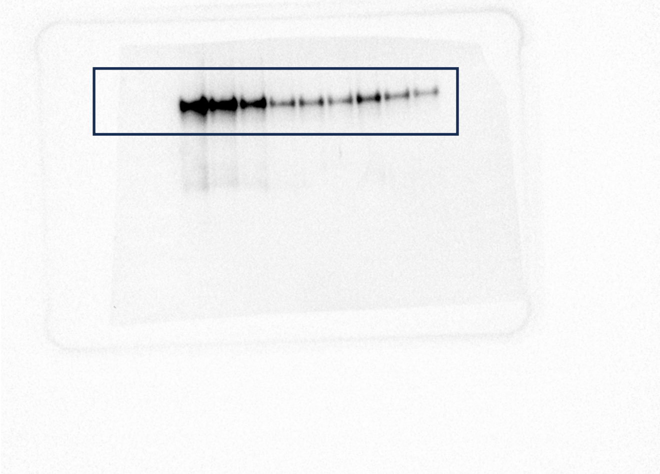

Supplement: SourceData F9 — is the source file for Fig. 9. [file jem_20221983_sourcedataf9.pdf]
